# Direct hydrodeoxygenation of raw woody biomass into liquid alkanes

**DOI:** 10.1038/ncomms11162

**Published:** 2016-03-30

**Authors:** Qineng Xia, Zongjia Chen, Yi Shao, Xueqing Gong, Haifeng Wang, Xiaohui Liu, Stewart F. Parker, Xue Han, Sihai Yang, Yanqin Wang

**Affiliations:** 1 Key Laboratory for Advanced Materials, Research Institute of Industrial Catalysis, East China University of Science and Technology, Shanghai 200237, China; 2 ISIS Facility, STFC Rutherford Appleton Laboratory, Chilton, Oxfordshire OX11 0QX, UK; 3 School of Chemistry, University of Nottingham, Nottingham, NG7 2RD, UK; 4 School of Chemistry, University of Manchester, Manchester M13 9PL, UK

## Abstract

Being the only sustainable source of organic carbon, biomass is playing an ever-increasingly important role in our energy landscape. The conversion of renewable lignocellulosic biomass into liquid fuels is particularly attractive but extremely challenging due to the inertness and complexity of lignocellulose. Here we describe the direct hydrodeoxygenation of raw woods into liquid alkanes with mass yields up to 28.1 wt% over a multifunctional Pt/NbOPO_4_ catalyst in cyclohexane. The superior performance of this catalyst allows simultaneous conversion of cellulose, hemicellulose and, more significantly, lignin fractions in the wood sawdust into hexane, pentane and alkylcyclohexanes, respectively. Investigation on the molecular mechanism reveals that a synergistic effect between Pt, NbO_*x*_ species and acidic sites promotes this highly efficient hydrodeoxygenation of bulk lignocellulose. No chemical pretreatment of the raw woody biomass or separation is required for this one-pot process, which opens a general and energy-efficient route for converting raw lignocellulose into valuable alkanes.

## Abstract (web summary)

The conversion of renewable lignocellulosic biomass into liquid fuels is attractive but extremely challenging due to the inertness and complexity of lignocellulose. Here, the authors describe the direct hydrodeoxygenation of raw woods into liquid alkanes over a multifunctional Pt/NbOPO_4_ catalyst.

## Graphical abstract

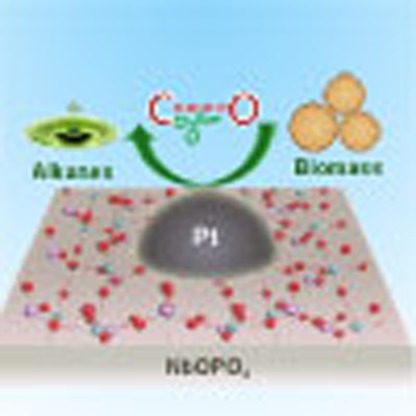

Fossil fuel consumption is projected to increase significantly in coming decades, with potentially catastrophic consequences for the environment^1^. Sustainable alternatives to crude oil are imperatively needed to bridge gaps in the supply of chemical fuels and feedstocks^2^. For the production of liquid fuels in particular, the replacement of oil-based routes by renewable biomass has received increasing attention^3^,^4^,^5^,^6^. Lignocellulose, as the main component of woody biomass, is composed of cellulose (40–50 wt%; a linear polymer of D-glucopyranose connected by *β*-1,4-glycosidic linkages), hemicellulose (16–33 wt%; a heteropolymer consisting of many different sugar monomers) and lignin (15–30 wt%; a heavily cross-linked, complex polymer with coumaryl, coniferyl and sinapyl alcohols as monomers)^7^,^8^. Owing to the complexity of lignocellulosic biomass and its notorious resistance to chemical transformation, energy-efficient and cost-effective production of liquid fuels from lignocellulose remains a mammoth challenge. So far, two strategies have been reported to address this challenge: (i) separation of lignocellulose into isolated sugars and lignin followed by biological or chemical (hydrolysis) processing^9^,^10^,^11^; (ii) thermochemical treatment of lignocellulose to produce upgradeable intermediates, such as bio-oils by pyrolysis or syngas by gasification, coupled with subsequent catalytic upgrading^12^,^13^. Thermochemical processes offer the total conversion of lignocellulose, but are often non-selective and intractable, and the resultant bio-oils or syngas need to be upgraded for further utilisation. Although hydrolysis-based approaches offer selective production of liquid fuels, they are generally multistep and thus very energy-intensive^14^. Moreover, the lignin by-products generated from the hydrolysis of lignocellulose are usually burned as a low-value fuel^15^. Powerful drivers therefore exist to develop alternative efficient and selective strategies to directly convert raw lignocellulose into liquid fuels.

Direct conversion of raw lignocellulose into alcohols and phenols was realised recently in exceptional cases^16^,^17^. However, successful direct production of hydrocarbon fuels (that is, total removal of oxygen) is mostly achieved so far from separated components of lignin or cellulose^18^,^19^,^20^. For example, the conversion of lignin into alkanes and methanol has been reported through a two-step process (chemical pretreatment and sequential hydrogenolysis and hydrogenation)^18^. Recently, the one-pot conversion of cellulosic feedstock into liquid alkanes in biphasic reaction systems (organic+water) were also reported over Ir–ReO_*x*_/SiO_2_–H–ZSM-5 or tungstosilicic acid–Ru/C catalysts^19^,^20^. The industrial Shell/GTI hydropyrolysis and Virent Energy System's approaches are also known to directly convert sugars or raw biomass into liquid fuels^21^,^22^. The former is based on a catalytic thermal–chemical technique, which reacts at very high temperature (350–540 °C) (ref. 21). Virent's approach converts water-soluble oxygenated hydrocarbons into C_4+_ hydrocarbons, alcohols and/or ketones in aqueous phase or vapour phase. This is achieved by aqueous phase reforming of water-soluble oxygenates, followed by condensation and deoxygenation^22^. More recently, a three-catalyst system was reported to convert raw biomass into liquid alkanes and other mono-functional hydrocarbons over layered LiTaMoO_6_ combined with Ru/C in aqueous phosphoric acid medium^23^. Gaining in-depth understanding on the reaction mechanism is of fundamental importance for the development of improved catalytic systems.

Here we report that, by using a multifunctional Pt/NbOPO_4_ catalyst, raw woody biomass can be directly converted into liquid alkanes in high yields in a single-phase medium (cyclohexane) with cellulose, hemicellulose and lignin fractions in solid woods being converted into hexane, pentane and alkylcyclohexanes, respectively (Fig. 1), representing direct conversion of raw lignocellulose into liquid alkanes under mild conditions over a single catalyst. Importantly, no chemical pretreatment (for example, hydrolysis and separation) to the raw wood is required for this process, and thus, tremendous energy savings can be potentially gained in comparison with the existing thermochemical- and hydrolysis-based approaches. More significantly, the pathway for this novel catalytic reaction was systematically investigated by control experiments, and the molecular mechanism for the rate-determining step in this conversion studied by *in situ* inelastic neutron scattering and computational studies. These complementary investigations reveal that the NbO_*x*_ species promotes the crucial C–O bond cleavage over hydrodeoxygenation of tetrahydrofuran (THF) and phenol (model units of cellulose and lignin, respectively) to hydrocarbons under mild reaction conditions.

## Results

### Direct hydrodeoxygenation of raw woody biomass

To verify the applicability of this one-pot approach, seven different types of wood sawdusts (<75 μm), including both softwoods and hardwoods, were employed as feedstocks for direct hydrodeoxygenation over the Pt/NbOPO_4_ catalyst in a cyclohexane medium (Table 1). The reactions were conducted at 190 °C and 5 MPa H_2_ for 20 h and over 20 wt% total mass yield of liquid alkanes was achieved for all woods, among which birch wood gave the highest mass yield of 28.1 wt%. Considering that the theoretical mass yield of alkanes from raw woody biomass is limited to ∼50 wt% as the removed oxygen accounts for almost half of the mass loss, the yields obtained here are excellent. In addition to C_1_–C_6_ alkane products, surprisingly appreciable amounts of alkylcyclohexanes (for example, propylcyclohexane and ethylcyclohexane) were also detected (Supplementary Fig. 1), indicating that not only the cellulose and hemicellulose but also the lignin fraction in sawdusts were converted into alkanes. Obviously, the source/texture of lignocellulose had a significant influence on both mass and carbon yields of the alkane products. In general, higher yields of hexanes and pentanes were achieved from softwoods: the carbon yields of hexanes and pentanes on the basis of cellulose and hemicellulose fractions reached 72.8 and 69.3% on average, respectively. These yields are surprisingly high and even comparable to those using isolated cellulose as feedstock^19^,^20^. Indeed, pure cellulose was tested as model material for carbohydrate fractions in raw woody biomass to confirm the performance of the catalyst. A total of 71.5% yield of hexanes and 8.7% yield of pentanes (by C–C cleavage) were achieved from cellulose conversion with excellent stability (Supplementary Table 1 and Supplementary Fig. 2). On the other hand, the yield of alkylcyclohexanes produced from hardwoods is much higher than that from softwoods, with an average carbon yield of 34.0% from hardwood (here only monomer alkylcyclohexanes were determined). It is worth noting that this yield is very high because there is a large proportion of C–C linkages in lignin structure (30–34% for hardwoods and 43–51% for softwoods on average)^15^, which are hardly cleaved under such mild reaction conditions, thus resulting in a maximum theoretical carbon yield of monomer alkylcyclohexanes at 44–49% from hardwoods and 24–32% from softwoods (Supplementary Note 1). This result indicates that the catalyst has excellent performance for the direct hydrogenolysis of C–O–C linkages of lignin and total hydrodeoxygenation of the resultant lignin monomers. To further confirm this, diphenyl ether and phenol, which possess aromatic ether and hydroxyl functionalities, respectively, were tested as model compounds over Pt/NbOPO_4_. A total of 99.9% yield of cyclohexane was achieved from both substrates, demonstrating the efficient cleavage of ether bond in lignin by this catalyst (Supplementary Table 1).

### Clarification of the unique activity of Pt/NbOPO_4_

A variety of catalysts with different combinations of support and metal (that is, Pt/NbOPO_4_, Pt/H–ZSM-5, Pt–ReO_*x*_/SiO_2_, Pt–ReO_*x*_/C and Pd, Ru, Rh loaded NbOPO_4_) were tested with birch sawdust as feedstock for this reaction to clarify the unique activity of Pt/NbOPO_4_ (Table 2). With Pt supported on H–ZSM-5, which posseses similar acidity to NbOPO_4_ but does not contain transition metal oxide (Supplementary Fig. 3), only 8.7 wt% yields of liquid alkanes was obtained (versus 28.1 wt% for Pt/NbOPO_4_). This suggests that the NbO_*x*_ species of NbOPO_4_ support has a significant promotion effect in this reaction. Such promotion effect on C–O cleavage was investigated recently in transition metal oxides of NbO_*x*_ and ReO_*x*_ (refs 24, 25, 26). To provide more insight, two other ReO_*x*_ support with reduced acidity, ReO_*x*_/SiO_2_ and ReO_*x*_/C, were tested for comparison, and alkane yields of 11.4 and 9.8 wt%, respectively, were obtained with a small amount of mono-functional hydrocarbons detected (for example, tetrahydropyran). This result suggests that the promotion effect should be accompanied by sufficient acidity to achieve efficient C–O cleavage in this reaction. The NbOPO_4_ support fulfills these two requirements (that is, surface NbO_*x*_ species and sufficient acidity), and thus posesses the best catalytic activity for this reaction. On the metal side, Pd, Ru and Rh were tested by loading them onto the NbOPO_4_ support, and moderate yields (17.2–19.2 wt%) of liquid alkanes were achieved from the hydrodeoxygenation of birch wood. The reason that Pt gave the highest yield among the studied metals is due to its supreme activity for H_2_ activation and hydrogenation (Supplementary Table 2). From these control experiments, we rationalise the superior performance of Pt/NbOPO_4_ by a synergistic effect between Pt, NbO_*x*_ species and the acidic sites on the support (including both Brönsted acid sites on PO_4_ and Lewis acid sites on NbO_*x*_, Supplementary Fig. 4).

### Study of the activity and stability of the catalyst

To investigate the applicability and recyclability of the catalyst, Pt/NbOPO_4_ was tested under various reaction conditions and the results are summerised in Table 3. As the products of this conversion were only alkanes, they act as an additional solvent to drive the sequential reactions without the need of solvent separation or recycling after each run. Cyclohexane was used as a solvent to facilitate the analysis because it does not overlap with any product. Alternatively, other alkane (for example, tridecane) can be used as a solvent for this reaction to afford similar results (Table 3, entry 1). Moreover, the reaction with double solid loadings was attempted, and there was no obvious decline of the alkane yields (Table 3, entry 2), indicating that this process is capable of dealing with higher solid loadings. Mineral poisoning is widely known to reduce the catalyst activity in biomass conversion. To probe more insight, extra ashes obtained by calcination of 1 g birch sawdust at 500 °C for 3 h were added into the system to test the cellulose conversion. The result suggests that a fivefold amount of ash has no influence on the catalytic activity of this catalyst (Table 3, entries 4 and 6). Moreover, the stability of the catalyst at lower alkane yields was tested by shortening the reaction time to 8 h. Small decreases on the yields of hexanes and pentanes were observed after four successive runs (hexanes: from 41.3 to 37.5%; pentanes: from 4.6 to 4.0%; Table 3, entries 6 and 7, Supplementary Fig. 2). Characterisations of the catalyst before and after the reaction showed small reductions on the BET surface area and the Pt dispersion (Supplementary Note 2), consistent with the observed small decreases on the yields of hexanes and pentanes. On the whole, the catalyst showed good and consistent catalytic performance in repeated runs in this one-pot process. This could be due to two reasons: first, the reaction was carried out under mild conditions (190 °C), which retards the significant aggregation of Pt particles (Supplementary Fig. 5); second, the use of non-aqueous single-phase medium (cyclohexane) hinders leaching and structural change of the catalyst (Supplementary Fig. 6). Indeed, the ICP analysis of the reaction solution suggested that the concentration of Pt, P or Nb was all below the detection limit, confirming the absence of catalyst leaching during the reaction.

### Studies of the representative reaction pathway

**Figure 2:**
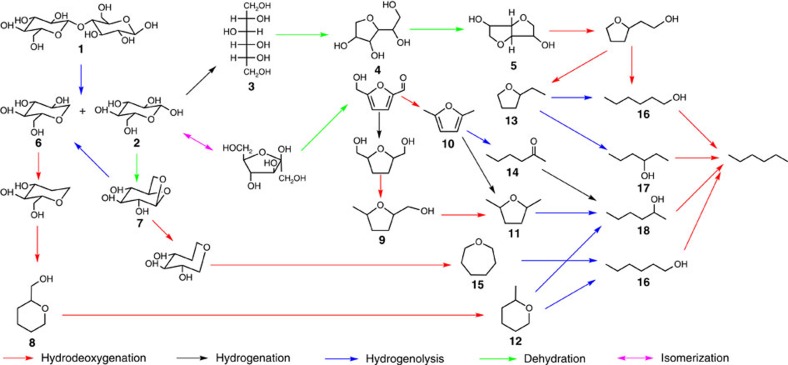
Representative reaction pathways for the direct hydrodeoxygenation of cellobiose. According to all the intermediates detected, it is possible to deduce the main reaction pathways of the direct conversion of cellobiose to hexane. The reaction occurred by the direct hydrogenolysis of the *β*-1,4 linkage to D-glucose (**2**) and 1-deoxy-D-glucose (**6**) first, and then **6** was converted to hexane by sequential hydrogenolysis via 2-hydroxymethyl-tetrahydropyran (**8**), 2-methyltetrahydropyran (**12**) and hexanols (**16**) and (**18**), whereas the conversion of **2** has three main reaction pathways: (i) hydrogenated to sorbitol (**3**) and then dehydrated to sorbitan (**4**) and isosorbide (**5**), followed by sequential hydrogenolysis via 2-ethyl-THF (**13**) and hexanols (**16**) and (**17**). (ii) Isomerised to fructose and then dehydrated to 5-hydroxymethylfurfural followed by hydrogenation and sequential hydrogenolysis via 2,5-dimethylfuran (**10**), 2-hexanone (**14**), 5-methyl-THF-2-methanol (**9**), 2,5-dimethyl-THF (**11**) and 2-hexanol (**18**). (iii) Dehydrated to 1,6-anhydroglucose (**7**) followed by sequential hydrogenolysis via oxepane (**15**) and *n*-hexanol (**16**).

In attempting to reveal the reaction pathway, model compounds were used to simplify the original reaction system for easier detection of intermediates and products. Diphenyl ether and phenol were chosen as the model compounds of the lignin fraction to investigate the cleavage of ether bond (as mentioned above), and cellobiose was used to represent the carbohydrate fraction to elucidate the conversion of cellulose and hemicellulose to alkanes. To better monitor the possible intermediates, the reaction was carried out at a lower temperature of 170 °C, and water was added into the reaction mixture after reaction to extract water-soluable intermediates. After 1 h reaction, small amounts of glucose (**2**), sorbitol (**3**), sorbitan (**4**) and isosorbide (**5**) were detected by HPLC (Supplementary Fig. 7), and a mixture of 1-dehydroxyl-glucose (**6**), 1,6 anhydro glucose (**7**), 2-hydroxymethyl-tetrahydropyran (**8**), 5-methyl-THF-2-methanol (**9**) as well as many other undefined intermediates in the aqueous phase were detected by GC–mass spectrometry (MS) (Supplementary Fig. 8). As no water existed at the beginning of this reaction, this result indicates that the *β*-1,4 linkage in cellobiose was cleaved by direct hydrogenolysis rather than hydrolysis, as a result of the excellent performance of Pt/NbOPO_4_ in hydrogenolysis. When further reacted for 6 h, appreciable amounts of hexane, 2,5-dimethylfuran (**10**), 2,5-dimethyl-THF (**11**), 2-methyltetrahydropyran (**12**), 2-ethyl-THF (**13**), hexanone (**14**), oxepane (**15**) and hexanols (**16–18**) were observed in the organic layer (Supplementary Fig. 9). In the aqueous phase, isosorbide (**5**) and 2-hydroxymethyl-tetrahydropyran (**8**) were the main residuals (Supplementary Fig. 10), indicating that **5** and **8** are the two major intermediates in cellulose conversion. When further reacted for 24 h, no residual was detected in the aqueous phase, and a large amount of **11–13** and **16–18** were observed in the organic phase, suggesting that the ring-opening of THF derivatives and the subsequent hydrodeoxygenation are the rate-determining steps (Supplementary Fig. 11). Therefore, the direct conversion of cellobiose to hexane occurs via the hydrogenolysis of the *β*-1,4 linkage to **2** and **6** first, which then undergo a combination of hydrogenolysis, dehydration, hydrogenation and isomerisation reactions. The main reaction pathways of cellobiose conversion were proposed in Fig. 2, and was further confirmed by the isolated reactions of the four main intermediates (Supplementary Fig. 12).

### Inelastic neutron scattering studies

As discussed above and previously reported^24^, the ring-opening and the sequential hydrodeoxygenation of THF derivatives (for example, 5-hydroxymethylfurfural) are problematic and often represent the major barrier for the conversion of bio-derived furans into alkanes. Indeed, it is the rate-determing step for this conversion. Direct visualisation of the interaction between adsorbed THF (as a model compund for various THF derivatives generated from this reaction; the use of THF instead of THF-derivitives gives a clearer interpretation of the results) and the catalyst surface is crucial to understand the molecular details of binding, ring-opening and hydrodeoxygenation of THF into alkanes (butane in this case). INS is a powerful neutron spectroscopy technique to investigate the dynamics of hydrogenous compounds by exploiting the high-neutron scattering cross-section of hydrogen (82.02 barns)^27^. In addition, INS is not subjected to any optical selection rules, and the calculation of INS spectra from DFT calculations is straightforward (Supplementary Note 3). Here, we have successfully combined *in situ* INS and DFT to investigate the vibrational properties of the THF–Pt/Nb_2_O_5_ system to reveal the mechanism of the challenging hydrodeoxygenation of THF. NbOPO_4_ has a large amount of surface P–OH groups, the vibrational peaks of which will overlap with signals of adsorbed THF. Therefore, we here used Pt/Nb_2_O_5_ instead for a clearer interpretation of the experimental observation. It is worth noting that Pt/Nb_2_O_5_ has a similar catalytic reactivity to Pt/NbOPO_4_ for this reaction under the same conditions, as evidenced by the direct comparison of the yield and selectivity data for THF conversion in Supplementary Table 3. To the best of our knowledge, this is the first example of using INS/DFT to study the mechanism of catalytic biomass conversion.

**Figure 3:**
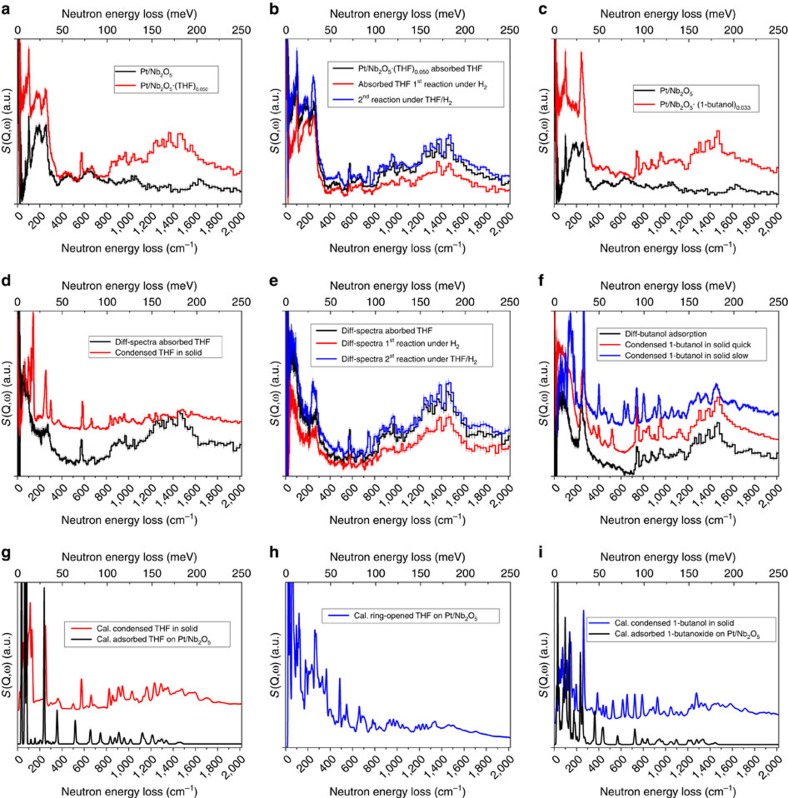
Inelastic neutron scattering spectra for Pt/Nb_2_O_5_ during the catalytic hydrodeoxygenation of THF. INS spectrum of the reduced catalyst was used throughout for calculations of the difference spectra. No abscissa scale factor was used throughout this report for INS calculations. NbOPO_4_ has a large amount of surface –OH groups, which have no specific activity in THF conversion, but will generate additional INS peaks overlapping with signals from substrates. Pt/Nb_2_O_5_ has a similar catalytic reactivity as Pt/NbOPO_4_ for the hydrodeoxygenation of THF and was therefore used in INS study instead. Comparison of the experimental INS spectra for bare catalyst and the THF (**a**) and 1-butanol (**c**) adsorbed catalyst. Comparison of the difference plots for experimental INS spectra of bare and THF (**b**) and 1-butanol (**f**) adsorbed catalyst, and the experimental INS spectra of condensed THF (**d**) and 1-butanol (**f**) in the solid state. (**b**) Comparison of the experimental INS spectra for the two hydrodeoxygenation reactions of adsorbed THF on the catalyst, and difference plots (with reduced catalyst as background) are shown in (**e**). The first reaction was carried out at 130 °C in pure H_2_ flow for 10 min and the second reaction was carried out at 130 °C in THF/H_2_ flow for 5 h, that is fresh THF vapour was fed into the reaction cell continuously with H_2_ as carrier gas during the reaction. (**g**) Comparison of the calculated INS spectra for condensed THF in solid and adsorbed THF on the catalyst. (**h**) View of the calculated INS spectrum for ring-opened THF on the catalyst. (**i**) Comparison of the calculated INS spectra for condensed 1-butanol in solid and adsorbed 1-butanoxide on the catalyst.

**Figure 4:**
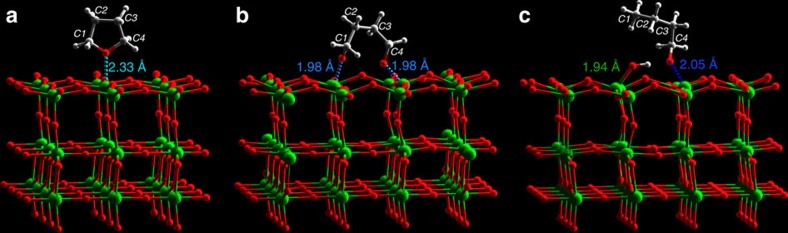
View of the optimised structural models. (**a**) adsorbed THF, (**b**) reacted (ring-opened) THF and (**c**) 1-butanol after adsorption to generate 1-butanoxide and surface hydroxyl on the (001) plane of the catalyst Pt/Nb_2_O_5_ (Nb: green; O: red; C; grey; H: white).

The INS spectrum of the bare catalyst gives a clean background with no prominent features (details on the discussion of background spectra are given in Supplementary Note 4 and Supplementary Figs 13 and 14). In comparison, the INS spectrum of the catalyst on THF adsorption at 130 °C shows a significant increase in total intensity, demonstrating the binding of THF to the catalyst surface (Fig. 3a). Comparison of the difference spectrum before and after THF adsorption on the catalyst (that is, signals for adsorbed THF) and that of the solid THF shows a few changes (Fig. 3d). Peaks at low energy (below 200 cm^−1^), assigned to the translational and rotational modes of THF, shift to lower energy with a continuum profile, suggesting that the adsorbed THF molecules are disordered over the catalyst surface, and have restricted translational motion owing to the strong binding to the catalyst. The very strong peak at 251 cm^−1^, assigned to the torsional mode of the C2–C3 bond of THF ring, reduced significantly in intensity, indicating the loss of this motion on adsorption. The peak at 300 cm^−1^, likely due to the combination of the lattice mode at ∼60 cm^−1^ and the strong mode at 251 cm^−1^, concurrently reduced in intensity. In addition, the ring deformation mode at 587 cm^−1^ in solid THF shifts to 571 cm^−1^ when adsorbed on the catalyst. A structural model of solid THF and THF adsorbed on Nb_2_O_5_ were optimised by DFT, respectively, and calculated INS spectra were produced (Fig. 3g; Supplementary Figs 15 and 16). Comparison of the INS spectra suggests that THF is likely adsorbed intact via interaction between its O(*δ*−) centre to open Nb(δ+) site (O···Nb=2.33 Å) on the surface (Fig. 4a) and that ring-opening of THF does not happen immediately on adsorption and/or in the absence of H_2_. The calculation also confirms the downshift of the ring deformation mode but slightly overestimates its magnitude.

The adsorbed THF underwent a first catalytic conversion in H_2_ flow for 10 min at 130 °C. The INS spectrum of the first reacted catalyst shows a large decrease in intensity (Fig. 3b,e), suggesting that the adsorbed THF underwent fast catalytic conversion to butane, which was sequentially swept out of the cell as confirmed by MS. In particular, the peaks at 1,244 and 1,308 cm^−1^ (assigned to –CH_2_– twisting and internal ring deformation of THF, respectively) disappeared completely, confirming the cleavage of the THF ring. An optimised structural model of ring-opened THF on Nb_2_O_5_ suggests that the adsorbed THF interacts with the very strong Lewis acid sites (Nb^5+^), and the ring opens via binding to two adjacent Nb^5+^ centres simultaneously (O···Nb=1.98 Å) (Figs 3h and 4b). Comparison of the calculated INS spectrum for ring-opened THF and experimental difference spectrum, however, does not conclusively suggest the presence of this intermediate bound on the catalyst. This could be due to two possible reasons: (i) the ring-opened intermediate is highly active and was hydrogenated instantly, and thus cannot be captured effectively; (ii) the amount of this intermediate is too low to be detected as no THF feedstock was provided during the reaction. To enrich the intermediate on the catalyst, a second catalytic reaction was conducted in THF/H_2_ flow for 5 h, and the production of butane observed continuously by MS. The INS spectrum of the second reacted catalyst indeed shows an increase in intensity even compared with that of THF-adsorbed catalyst, confirming the presence of additional substrates on the catalyst surface (Fig. 3b). The corresponding difference spectra confirm the presence of adsorbed THF, and more importantly, four new peaks at 245, 477, 744 and 805 cm^−1^ were observed (Fig. 3e), indicating the presence of additional hydrogenous species bound on the catalyst.

To reveal the identity of this intermediate/s, the adsorption of 1-butanol on the catalyst was studied because 1-butanoxide bound to Nb^5+^ is predicted to be relatively stable (Fig. 3i). The INS spectrum of 1-butanol adsorbed on the catalyst shows a large increase in intensity, and comparison of the difference spectrum (that is, signals for adsorbed 1-butanol) and that of solid 1-butanol shows significant changes (Fig. 3c,f). The most important one is the disappearance of the peak at 839 cm^−1^ (assigned to the out-of-plane C–O–H bending mode, detailed discussions are given in Supplementary Note 4 and Supplementary Fig. 17), clearly suggesting 1-butanol underwent deprotonation on adsorption to give 1-butanoxide bound to the surface Nb^5+^ sites (Fig. 4c and Supplementary Fig. 18). The conformation-dependent bands between 300 and 600 cm^−1^ in the INS spectrum of solid 1-butanol all disappeared on adsorption, indicating that the alkyl chain of adsorbed 1-butanoxide is not in the all-*trans* conformation as found in crystalline 1-butanol (Supplementary Note 4). In addition, the peak at 257 cm^−1^ (assigned to the methyl torsion) shifts to lower energy at 243 cm^−1^ on binding to the catalyst. Moreover, an additional peak at 1,265 cm^−1^ (assigned to C4 chain deformation) is present for bound 1-butanoxide on Nb^5+^.

Comparison of the INS spectra of adsorbed 1-butanoxide and the second reacted catalyst gives a clear message: three out of the four new peaks at 245, 744, and 805 cm^−1^ (assigned as methyl torsion, –CH_2_CH_2_– rocking and –CH_2_CH_2_CH_3_ rocking of 1-butanoxide, respectively) of the second reacted catalyst are consistent with the presence of 1-butanoxide bound on the surface (Fig. 3e,f). The remaining new peak at 477 cm^−1^ is conformation-dependent and consistent with the presence of a *gauche* conformer of C4 chain of bound 1-butanoxide^28^. A final INS spectrum for the reactivated catalyst shows no prominent feature (Supplementary Fig. 19), confirming the absence of formation of residual hydrocarbonaceous species and thus demonstrating the high efficiency of catalyst regeneration in cycling experiments.

The calculated and experimental INS spectra for solid THF and 1-butanol show excellent agreement; however, those for the guest-bound-catalysts exhibit a number of discrepancies (Fig. 3d,i). It is worth noting that the experimental data were collected on a disordered system (poorly crystalline mesoporous metal oxide and disordered substrates), wheras the calculations assume fully periodic structures. A poorly crystalline system generates multiple sites with different binding energies, inducing the broadening of INS peaks. The presence of different conformers of the butane chain on the catalyst also induces discrepancy as the calculation was done with the *gauche* conformer with the lowest energy only and the low energy modes are very sensitive to the chain conformation. Indeed, the INS spectra for disordered and crystalline 1-butanol have suggested that the crystallinity of the system can induce significant changes to both peak intensity and positions (Supplementary Fig. 17). Moreover, the calculation used a model with “flat” surface, in which there is no interaction with the surface, other than through the THF/1-butanol oxygen atom. On the real, rough surface, for example, with steps (Supplementary Fig. 5), the hydrogen atoms on C1 and C4 of THF (or C1 and C2 of 1-butanol) will also be enabled to interact with the surface, further enhancing the binding and broadening of the INS bands.

In this study, we concentrate primarily on the experimental observation, which has confirmed that (i) adsorbed THF molecules on the catalyst have, in principle, an intact structure with reduced motion (esp. for the C2–C3 torsion and ring deformation modes) owing to the strong binding to the Nb^5+^ sites; (ii) ring-opening of adsorbed THF (cleavage of C–O bond) occurs rapidly in the presence of H_2_ as shown by the loss of the internal THF ring deformation mode; (iii) 1-butanoxide bound to the surface Nb^5+^ site is a relatively stable reaction intermediate, consistent with calculations (see below). Therefore, this catalytic conversion of THF follows adsorption, binding, ring-opening, partial hydrogenation and complete hydrodeoxygenation, and the surface Nb^5+^ sites played an important role in this reaction, particularly for the binding and activation of THF substrates. It is worth noting that here the role of Pt is believed to dissociate H_2_ and provide [H], and such role is not exclusive in the rate-determining C–O–C bond cleavage step, considering that similar performance can be obtained by substituting Pt with Pd loaded on NbOPO_4_ for the hydrodeoxygenation of cellulose into alkanes (Supplementary Table 3). However, other possible roles of Pt in the whole reaction such as strengthening acidity through interface interactions^29^ may not be fully ruled out, which is beyond the core point of this work and will be studied further in future works.

### Computational studies of the catalytic origin of NbOPO_4_

To probe more insight, first-principle calculations were conducted to examine the crucial C–O bond breaking process of phenol and 1-butanol, which are the model compound of lignin and important intermediate in THF conversion, respectively. Notably, the widely used ReO_*x*_ catalyst in biomass conversion was also tested for comparison. The well-ordered flat NbOPO_4_(100) and Re_2_O_7_(010) surfaces, which have exposed five-coordinated Nb_5c_ and Re_5c_ centres as the main binding sites, were chosen as the substrates (Supplementary Methods, Supplementary Figs 20,21 and Supplementary Table 4).

**Figure 5:**
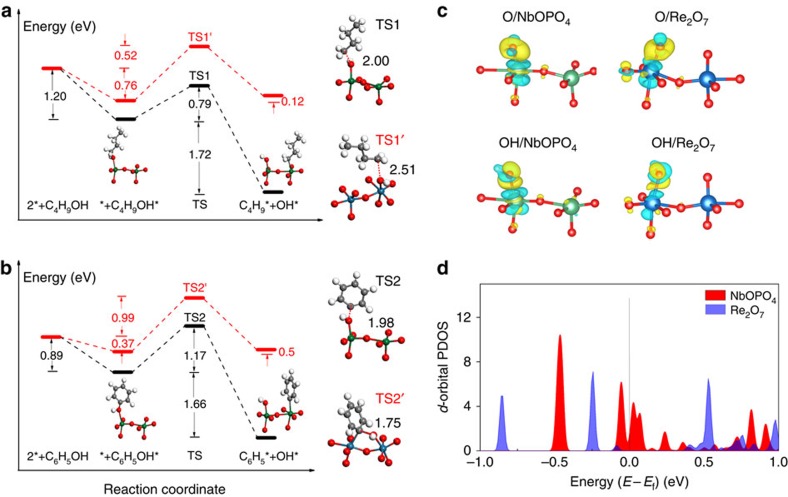
Calculated results of computational studies. (**a**,**b**) Calculated energy profiles of C–O bond cleavage of C_4_H_9_OH and C_6_H_5_OH, respectively. Black and red lines indicate NbOPO_4_(100) and Re_2_O_7_(010), respectively. The structures of the initial and final states on NbOPO_4_(100) are shown, whereas the transition states involving C–O bond cleavage of C_4_H_9_OH and C_6_H_5_OH are depicted in TS1, TS1′,TS2 and TS2′, with the elongated C–O bond lengths shown. Emerald balls represent Nb atoms, dark blue for Re, white for H, grey for C and red for O. (**c**) The isosurfaces of charge density difference for O and OH adsorption on NbOPO_4_(100) and Re_2_O_7_(010) surfaces. For clarity, only the local active sites are shown. The regions depicted in yellow indicate charge accumulation and light blue for charge depletion. Emerald balls represent Nb atoms, dark blue for Re, white for H and red for O. (**d**) The *d*-orbital projected density of states for the surface Nb_5c_ and Re_5c_ atoms, demonstrating their relative energies, in which the energy is aligned to the Fermi level (*E*_F_).

For 1-butanol conversion on NbOPO_4_, the calculation indicates that 1-butanol efficiently adsorbs on Nb_5c_ with a corresponding adsorption energy of −1.20 eV, which is evidently stronger than that on Re_2_O_7_ (−0.76 eV), indicating that NbOPO_4_(100) possesses a stronger binding ability. Subsequently, with the aid of the surface Nb_5c_, the C–O bond can break with each OH and butyl occupying a Nb_5c_ site as the product; this process is strongly exothermic by 1.72 eV and gives a barrier of only 0.79 eV (Fig. 5a), implying its feasibility in both thermodynamics and kinetics (Supplementary Note 5 and Supplementary Table 5). In contrast, on Re_2_O_7_, 1-butanol dissociation is an endothermic process and has to overcome a larger barrier of 1.28 eV (Supplementary Note 5). By comparing the energy profiles (Fig. 5a), it is conclusive that NbOPO_4_ demonstrates an inherently better performance for 1-butanol deoxygenation than Re_2_O_7_. Likewise, with respect to C–O bond cleavage of phenol, we performed the same calculation, which yields a similar conclusion that NbOPO_4_ catalyses phenol dissociation more efficiently with a lower barrier than Re_2_O_7_ (Fig. 5b and Supplementary Note 5). Therefore, the high catalytic activity of NbOPO_4_ can be ascribed to better adsorption capability of surface Nb_5c_ and a lower activation barrier in comparison to Re_2_O_7_. Correlation between the C–O bond dissociation barriers and their corresponding adsorption energies (Supplementary Fig. 22) shows that stronger the bond strength of M_5c_–O (M=Nb, Re) leads to easier the C–O bond cleavage, which is in line with the principle of Brønsted–Evans–Polanyi relationship^30^,^31^,^32^. In other words, the outstanding binding ability of NbOPO_4_ is one determining factor in the efficient deoxygenation of cellulose and lignin. We also calculated and compared the adsorption energies of NbOPO_4_ with two other typical catalysts (Re_2_O_7_, ZrO_2_) toward various intermediate species, such as OH, O, THF, butyl and butoxy (Supplementary Table 6). Indeed, NbOPO_4_ demonstrates the strongest binding ability seamlessly, whereas ZrO_2_, with the weakest adsorption, is rarely applied for cellulose conversion to alkane in practice.

We are now in a position to elucidate the inherent mechanism of NbOPO_4_ exhibiting such an exceptional binding ability. An electronic structure analysis was made to understand the bond properties of M_5c_–O(OH) and the energy level of surface M_5c_. The isosurface of charge density difference shows electron accumulation between O (or OH) and Nb_5c_ (or Re_5c_) (Fig. 5c), indicating a typical covalent bond character. Therefore, the binding strength with O(OH) is mainly decided by the size and energy level of the *d* orbital of Nb_5c_ and Re_5c_ cations. The projected density of states on the *d*-orbitals of surface Nb_5c_ and Re_5c_ indicates that the *d* band of Nb_5c_ near the Fermi level (*E*_F_) is more delocalised comparing to Re_5c_, and more importantly, the energy level of the highest occupied *d* bands were evidently higher for Nb_5c_ with some metallic character across *E*_F_ (Fig. 5d). According to the frontier orbital theory, from these *d* bands features one can rationalise such a strong binding ability of NbOPO_4_ in facilitating deoxygenation reaction.

## Discussion

We have presented a one-pot catalytic process for the direct production of liquid alkanes from a wide variety of raw woody biomass over Pt/NbOPO_4_ catalyst with excellent mass and carbon yields in a cyclohexane medium. This one-pot approach avoids the separation of raw biomass into isolated components and the use of an alkane solvent further simplifies downstream separation, as the alkane products can be used as solvents for the next run. The exceptional activity of the Pt/NbOPO_4_ catalyst enabled direct conversion of raw woody biomass into liquid alkanes under mild conditions (190 °C) over a single multifunctional catalyst. The superior efficiency of this catalyst for direct hydrodeoxygenation of lignocellulose is found to originate from the synergistic effect between Pt, NbO_*x*_ species and acidic sites. This brand new one-pot route requires no chemical pretreatment or separation of the raw woody biomass and thus tremendous energy savings can be potentially gained in comparison to the existing thermochemical and hydrolysis-based approaches for production of liquid fuels and chemical feedstocks from lignocellulose.

## Methods

### Catalyst preparation

NbOPO_4_ used here was synthesised by a hydrothermal method at pH=2 according to literature^33^. M/NbOPO_4_ (M=Pt, Pd, Rh, Ru) catalysts were prepared by incipient wetness impregnation of NbOPO_4_ with aqueous solutions of Pt(NO_3_)_2_, Pd(NO_3_)_2_, Rh(NO_3_)_3_ and RuCl_3_, respectively. After impregnation, the catalysts were dried at 100 °C for 12 h, followed by calcination in air at 500 °C for 3 h. Pt/H–ZSM-5 was prepared by the same procedure. Pt–ReO_*x*_/SiO_2_ and Pt–ReO_*x*_/C were prepared by sequential incipient wetness impregnation. Pt was loaded onto the support first, dried and calcined before the process was repeated for Re. The loading of Pt, Pd, Rh, Ru was 5 wt% in all cases, for Pt–ReO_*x*_/SiO_2_ and Pt–ReO_*x*_/C, the loading amount of Re was 1 by the molar ratio of Re to Pt. The dispersion of Pt/NbOPO_4_ catalysts with different Pt loading amount was presented in Supplementary Fig. 23.

### Reaction system and product analysis

The direct hydrodeoxygenation of wood sawdusts was conducted in a 50 ml Teflon-lined stainless-steel autoclave. In a typical run, feedstock (0.20 g), catalyst (0.20 g) and cyclohexane (6.46 g) were put into the reactor, which was then sealed, purged three times with H_2_ and charged to an initial pressure of 5.0 MPa with H_2_. The reactor was then slowly heated to 190 °C under vigorous stirring and held at this temperature for 20 h. After the reaction finished, the reactor was quenched in an ice/water bath. The gas phase was carefully collected in a gas bag and analysed by GC equipped with a packed column, a methaniser (for CO_2_ detection) and a flame ionisation detector (FID). The products in the liquid phase were qualitatively analysed by GC–MS and quantitatively analysed by GC–flame ionisation detector equipped with a HP-5 column. The yields of liquid alkanes were determined by adding dodecane as an internal standard after reaction. Mass yields were calculated by the equation: mass yield of pentanes (hexanes, alkylcyclohexanes)=[mass of pentanes (hexanes, alkylcyclohexanes)]/[mass of feedstock input]. Carbon yields were calculated by the equation: carbon yield of pentanes (hexanes, alkylcyclohexanes)=[mass of carbon in pentanes (hexanes, alkylcyclohexanes)]/[mass of carbon in hemicellulose (cellulose, lignin)].

### Reaction pathways study

The direct hydrodeoxygenation of cellobiose was carried out in a similar way to that of wood sawdusts. After the reaction was quenched in an ice/water bath, 6.5 g of water was added into the autoclave under vigorous stirring for a few minutes to extract the hydrophilic intermediates and to dissolve cellobiose for total analysis. The aqueous phase was analyzed by HPLC (Agilent 1200 series) equipped with a Shodex SUGAR SC-1011 column and a differential refractive index detector and by GC–MS to monitor the intermediates and unreacted cellobiose. The organic phase was analysed by GC–MS to observe the lipophilic intermediates.

### Neutron scattering experiments

INS spectra were recorded on the TOSCA spectrometer at the ISIS Facility at the STFC Rutherford Appleton Laboratory (UK). TOSCA is an indirect geometry crystal analyser instrument that provides a wide dynamic range (16–4,000 cm^−1^) with resolution optimised in the 50–2,000 cm^−1^ range^34^. In this region TOSCA has a resolution of 1.25% of the energy transfer. The Pt/Nb_2_O_5_ (34.7 g) catalyst was loaded into an *in situ* catalysis cell with a copper vacuum seal and connected to a gas handling system. The sample was heated at 300 °C (5 °C/min ramping) under He for 3 h to remove any remaining trace water before the experiment. The sample was cooled to room temperature and a weight loss of 0.1 g was noted, assigned to loss of adsorbed water. The samples were cooled to <15 K during data collection by a closed cycle refrigerator cryostat. The procedure of the *in situ* catalysis experiment with the INS measurements is summarised in Supplementary Fig. 24. INS spectra for condensed THF (2.31 g) and 1-butanol (2.36 g) in the solid state were measured in a flat-plate sample container below 12 K. INS spectra for condensed THF and 1-butanol were used to (i) calculate the amount of adsorbed THF and 1-butanol onto the catalyst in each case by the integration of the peak areas; (ii) identify and compare the vibrational modes for the adsorbed and the free molecules.

Estimation of the amount of adsorbed THF on the catalyst: on the basis of the relative intensities of the INS peak at ∼600 cm^−1^ and the known mass (2.31 g) of THF in the condensed sample, there is 0.42 g of adsorbed THF present on the catalyst in the neutron beam.

Estimation of the amount of adsorbed 1-butanol on the catalyst: on the basis of the relative intensities of the INS peak at ∼740 cm^−1^ and the known mass (2.36 g) of 1-butanol in the condensed sample, there is 0.30 g of adsorbed 1-butanol present on the catalyst in the neutron beam.

Adsorption of THF was carried out by flowing THF vapour (∼200 mbar) in He (1.1 bar, 0.2 l min^−1^; this flow condition was used throughout the study) over the catalyst at 130 °C for 3 h. The cell was then flushed briefly with pure He flow for 2 min to remove the free and weakly bound THF on the catalyst, sealed and cooled to below 15 K for INS data collection. The adsorbed THF underwent the first catalytic conversion in pure H_2_ flow for 10 min at 130 °C, and production of butane was observed instantly by mass spectrometry. The cell was then flushed with He to remove free butane and H_2_, sealed and cooled again for INS collection to detect the presence of possible reaction intermediates.

## Additional information

**How to cite this article**: Xia, Q. *et al.* Direct hydrodeoxygenation of raw woody biomass into liquid alkanes. *Nat. Commun.* 7:11162 doi: 10.1038/ncomms11162 (2016).

## Supplementary Material

Supplementary Information Supplementary Figures 1-24, Supplementary Tables 1-6, Supplementary Notes 1-5, Supplementary Methods and Supplementary References

## Figures and Tables

**Figure 1:**
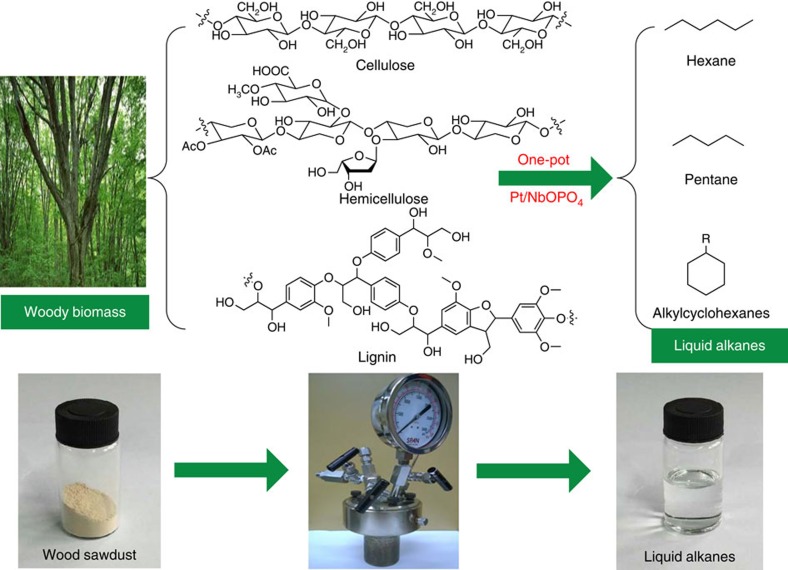
Schematic representation of direct hydrodeoxygenation of raw woody biomass into liquid alkanes. Raw woody biomass can be directly converted into liquid alkanes over Pt/NbOPO_4_ catalyst in cyclohexane medium, with cellulose, hemicellulose and lignin fractions in solid woods being converted into hexanes, pentanes and alkylcyclohexanes, respectively.

**Table 1:** Summary of direct hydrodeoxygenation of various woody biomass over Pt/NbOPO_4_.*

Woody biomass	Softwood				Hardwood		
	**Pine**	**White pine**	**Larch**	**Fir**	**Camphor**	**Birch**	**Poplar**
*Lignocellulose content*							
Hemicellulose (wt%)	9.3	10.4	13.1	10.3	24.8	25.7	28.2
Cellulose (wt%)	47.2	52.3	49.4	43.5	45.1	48.7	45.3
Lignin (wt%)	32.6	29.5	28.3	33.9	22.6	20.4	22.8

*Mass yield/carbon yield (wt%/mol%)*†							
Pentanes‡	4.1/80.8	3.6/63.4	4.8/67.2	3.7/65.9	7.3/54.0	10.2/73.1	7.1/46.2
Hexanes§	18.7/74.8	19.3/69.4	20.4/77.7	16.0/69.2	15.2/63.4	13.1/50.7	9.7/40.3
Alkylcyclohexanes||	2.1/9.6	2.2/11.2	2.6/13.7	2.3/10.1	5.1/33.7	4.8/35.1	5.1/33.3
Total liquid alkanes (wt%)	24.9	25.1	27.8	22.0	27.6	28.1	21.9
Others (wt%)¶	1.7	1.7	2.0	1.4	1.9	2.3	1.6
Residue (wt%)	31.5	27.9	27.1	33.4	17.4	14.8	16.7

^*^The reactions were conducted at 190 °C and 5 MPa H_2_ for 20 h. Feedstock (0.20 g), Pt/NbOPO_4_ (0.20 g) and cyclohexane (6.46 g) were put into a 50 ml stainless-steel autoclave.

^†^Mass yields were calculated by the equation: mass yield of pentanes (hexanes, alkylcyclohexanes)=[mass of pentanes (hexanes, alkylcyclohexanes)]/[mass of feedstock input]. Carbon yields were calculated by the equation: carbon yield of pentanes (hexanes, alkylcyclohexanes)=[mass of carbon in pentanes (Hexanes, alkylcyclohexanes)]/[mass of carbon in hemicellulose (cellulose, lignin)].

^‡^Pentanes include *n*-pentane and iso-pentane.

^§^Hexanes include *n*-hexane and iso-hexane.

^||^Only monomer alkylcyclohexanes were determined here, including methylcyclohexane, ethylcyclohexane, isopropylcyclohexane and propylcyclohexane.

^¶^Others were mainly CO_2_ and C_1_–C_4_ alkanes.

**Table 2:** The results of direct hydrodeoxygenation of birch wood over different catalysts.*

**Entry**	**Catalyst**	**Mass yield of liquid alkanes (wt%)**				
		**Pentanes**†	**Hexanes**‡	**Alkylcyclohexanes**§	**Total**	**Residue**
1	Pt/NbOPO_4_	10.2	13.1	4.8	28.1	14.8
2	Pt/H–ZSM-5	4.9	1.3	2.5	8.7	35.8
3	Pt–ReO_*x*_/SiO_2_||	5.2	3.6	2.6	11.4	26.9
4	Pt–ReO_*x*_/C||	6.4	1.5	1.9	9.8	ND
5	Pd/NbOPO_4_	8.1	6.2	3.5	17.8	29.4
6	Ru/NbOPO_4_	7.3	8.0	3.9	19.2	36.3
7	Rh/NbOPO_4_	8.2	5.0	4.0	17.2	31.5

^*^The reactions were conducted at 190 °C and 5 MPa H_2_ for 20 h. Feedstock (0.2 g), catalyst (0.2 g), and cyclohexane (6.46 g) were put into a 50 ml stainless-steel autoclave. All metal loading was 5 wt%.

^†^Pentanes include *n*-pentane and iso-pentane.

^‡^Hexanes include *n*-hexane and iso-hexane.

^§^Only monomer alkylcyclohexanes were determined, including methylcyclohexane, ethylcyclohexane, isopropylcyclohexane and propylcyclohexane.

^||^The loading amount of Re was 1 by the molar ratio of Re to Pt.

**Table 3:** Summary of direct hydrodeoxygenation of birch sawdust and cellulose under various reaction conditions over Pt/NbOPO_4_.*

**Entry**	**Feedstock**	**Carbon yield of liquid alkanes (%)**		
		**Hexanes**†	**Pentanes**‡	**alkylcyclohexanes**
1§	Birch sawdust	72.4	52.3	34.6
2||	Birch sawdust	69.1	49.7	35.9
3	Cellulose	71.5	8.7	ND¶
4#	Cellulose	40.3	4.4	ND
5**	Celloluse	65.2	8.2	ND
6††	Cellulose	41.3	4.6	ND
7††‡‡	Celloluse	37.5	4	ND

^*^Unless otherwise specified, the reactions were conducted at 190 °C and 5 MPa H_2_ for 20 h. Feedstock (0.2 g), catalyst (0.2 g), and cyclohexane (6.46 g) were put into a 50 ml stainless-steel autoclave.

^†^Hexanes include *n*-hexane and iso-hexane.

^‡^Pentanes include *n*-pentane and iso-pentane.

^§^Tridecane was employed as the reaction solvent.

^||^0.4 g of birch sawdust, 0.4 g of catalyst, and 6.46 g of cyclohexane were put into a 50 ml stainless-steel autoclave.

^¶^“ND” is the abbreviation of “not determined”.

^#^Ashes (minerals) obtained by calcination of 1 g birch sawdust at 500 °C for 3 h were added into the reaction system and reaction for 8 h.

^**^The cellulose was unmilled and used directly.

^††^The reaction time was 8 h.

^‡‡^Data shown are for the fourth run of the stability test.
