# Supplementary material for: Direct hydrodeoxygenation of raw woody biomass into liquid alkanes
Source: Nat Commun. 2016 Mar 30;7:11162. doi: 10.1038/ncomms11162 (PMC4820995; doi:10.1038/ncomms11162)
Supplement: Supplementary Information — Supplementary Figures 1-24, Supplementary Tables 1-6, Supplementary Notes 1-5, Supplementary Methods and Supplementary References [file ncomms11162-s1.pdf]

Supplementary Information

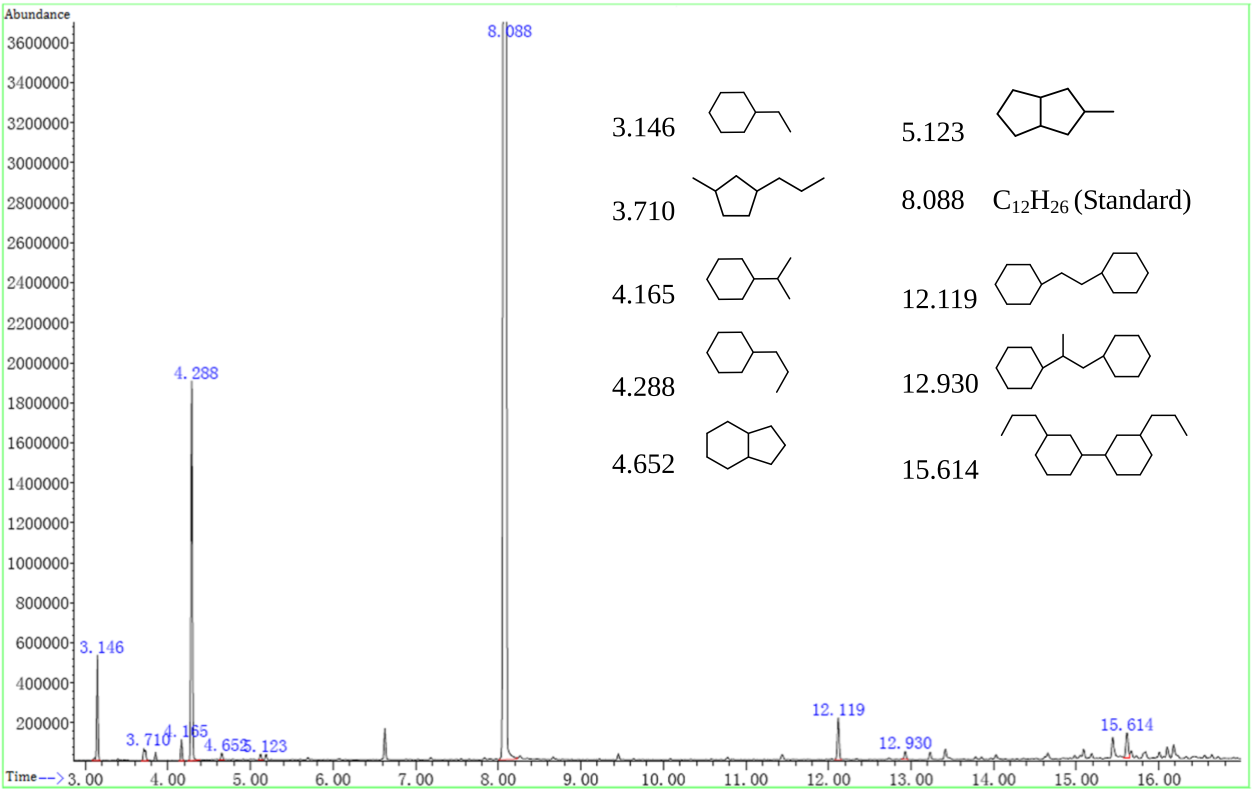

Supplementary Figure 1: Typical GC-MS spectra of the reaction effluent obtained from direct conversion of the lignin component of birch wood.

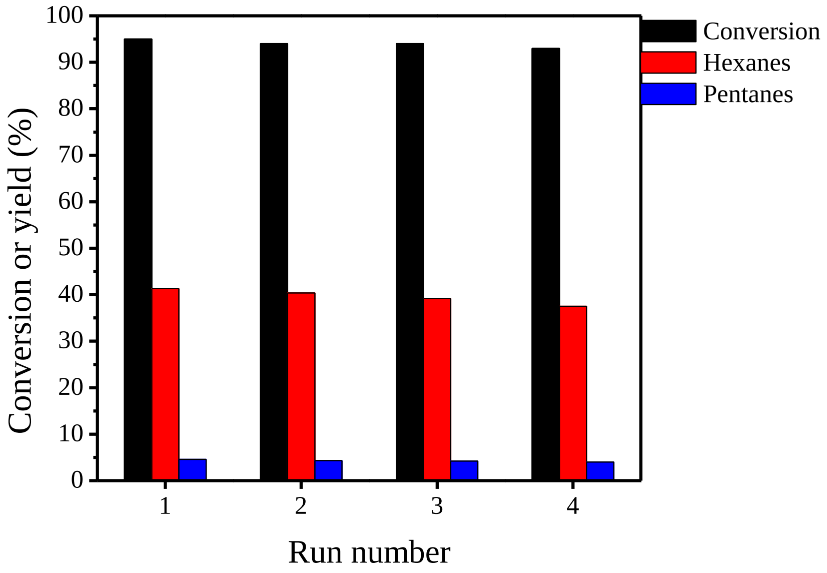

Supplementary Figure 2: Recyclability tests of Pt/$NbOPO_4$ catalyst for the direct hydrodeoxygenation of cellulose into liquid alkanes. The recyclability of the Pt/$NbOPO_4$ catalyst was investigated at 190 ^oC and 5 MPa initial H_2 pressure for 8 h using cellulose as feedstock. After each reaction cycle, the catalyst was first separated from the liquid phase by centrifugation, then dried and regenerated for the next run. The XRD patterns of the fresh and spent Pt/$NbOPO_4$ showed that the $NbOPO_4$ support remained amorphous after reaction (Supplementary Fig. 6). The specific BET surface area of Pt/$NbOPO_4$ decreased from 217 m^2 g^{-1} (before reaction) to 156 m^2 g^{-1} after reaction, likely due to the loss of some micropores after the reaction. The CO adsorption measurement showed that the dispersion of Pt on $NbOPO_4$ support decreased from 50.9% to 29.6%, due to the slow growth of the Pt particle and decrease of the specific area of the support. Nevertheless, the decreases of the microporosity and Pt dispersion have little influence on the activity of the catalyst in this reaction within four cycles.

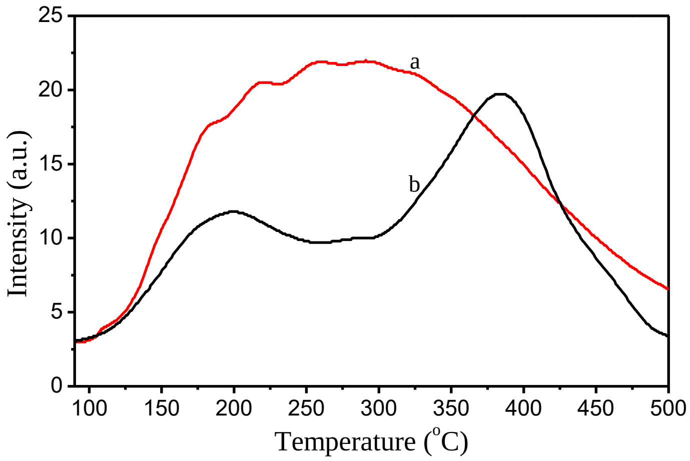

Supplementary Figure 3: The NH_3-TPD curves obtained for $NbOPO_4$ (a) and H-ZSM-5 (b). The NH_3-TPD was carried out in a chemisorption analyzer (PX200) equipped with a thermal conductivity detector (TCD). The samples (100 mg) were loaded into a U-shaped quartz tube. Prior to TPD measurements, the samples were pretreated in flowing N_2 (45 ml min^{-1}) for 1 h at 500 °C, and then cooled to 50 °C. NH_3 was adsorbed onto the samples by exposure to a flowing 10% NH_3 in N_2 gas mixture (50 ml min^{-1}) for 45 min at 50 °C. Residual and physically adsorbed NH_3 were removed by purging the samples with flowing N_2 (45 ml min^{-1}) at 90 °C for 1 h. Desorption of NH_3 was performed by heating the samples at a rate of 10 °C min^{-1} under flowing N_2 (45 ml min^{-1}) from 90 °C to 550 °C. The resulted NH_3-TPD profile shows that $NbOPO_4$ (a) has a broad symmetrical peak centered at ca. 275 °C, which can be assigned to desorption from medium to strong acid sites of $NbOPO_4$. H-ZSM-5 (b) has two peaks centered at 200 °C and 390 °C, which can be assigned to desorption from weak acid sites and strong acid sites, respectively. The NH_3-TPD measurements of $NbOPO_4$ and H-ZSM-5 show these two supports possess similar numbers of acid sites.

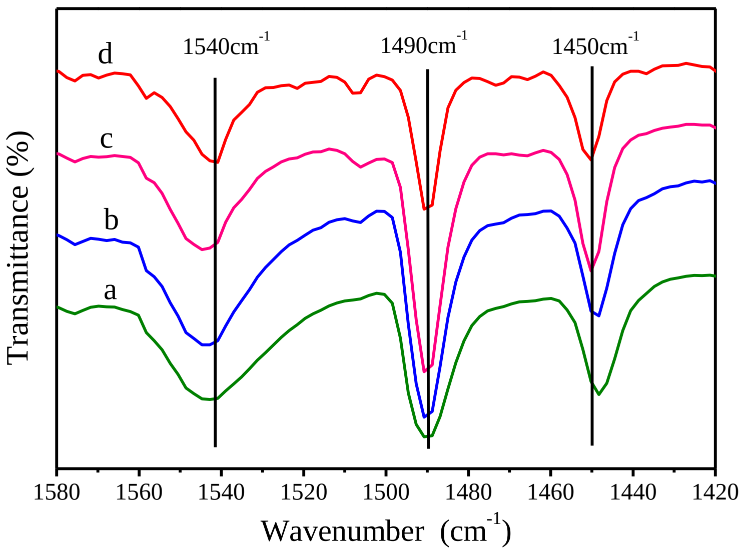

Supplementary Figure 4: Pyridine-FTIR spectra of $NbOPO_4$ obtained after evacuation at different temperatures. (a) 100 °C (b) 200 °C (c) 300 °C and (d) 400 °C. The band at 1490 cm^{-1} can be attributed to the adsorption of pyridine at Brönsted and Lewis acid sites at the same time. The band at 1450 cm^{-1} corresponds to the adsorption of pyridine at the Lewis acid sites and the band at 1540 cm^{-1} is the characteristic of the adsorption of pyridine at Brönsted acid sites. This result indicates that $NbOPO_4$ possesses both Brönsted and Lewis acid sites.

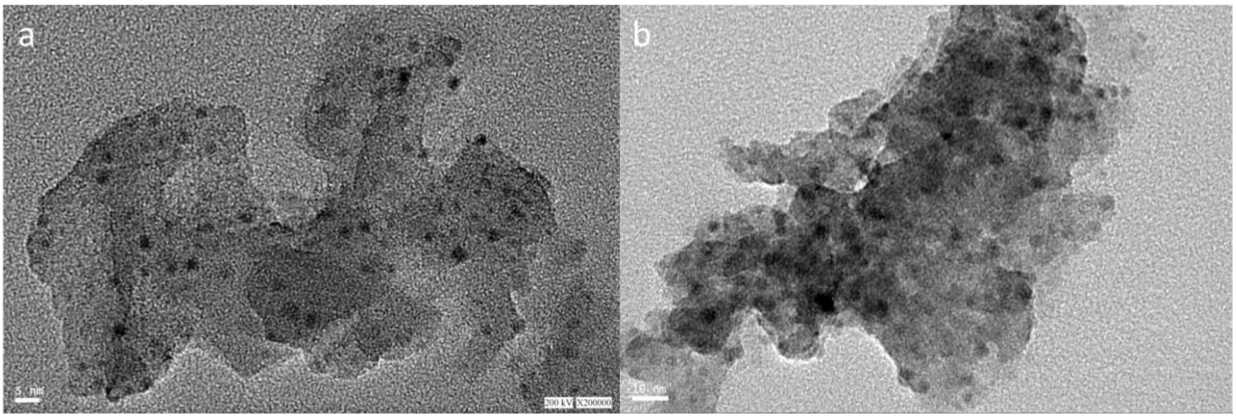

Supplementary Figure 5: TEM images of fresh (a) and spent (b) 5 wt% Pt/$NbOPO_4$. TEM images of fresh and spent Pt/$NbOPO_4$ confirmed the absence of significant aggregation of Pt particles after the reaction. However, the average size of Pt particles increased slightly due to the regeneration process which required the calcination of the catalyst at 500 ^oC.

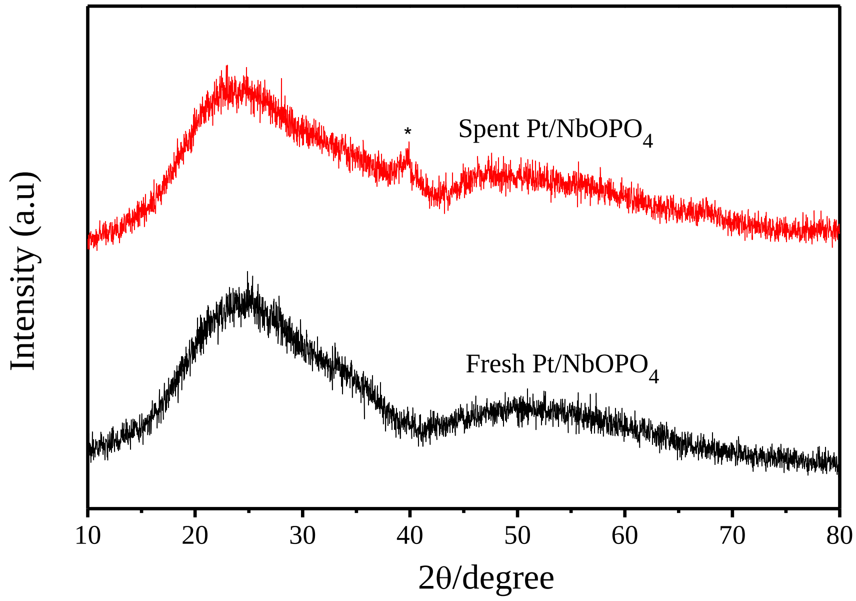

Supplementary Figure 6: XRD patterns of the fresh and spent Pt/NbOPO$_4$. The small broad peak at 2θ = 40.3 is attribute to the formation of a small amount of PtO_2 particles, which is in accordance with the TEM and CO adsorption results.

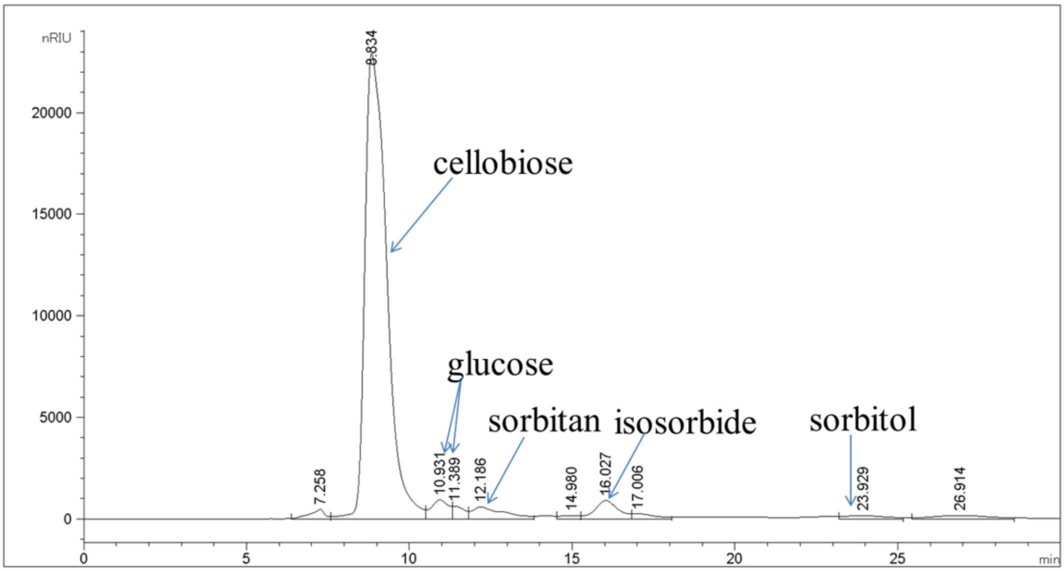

Supplementary Figure 7: HPLC spectra of reaction effluent obtained after cellobiose reacting at 170 °C for 1 h.

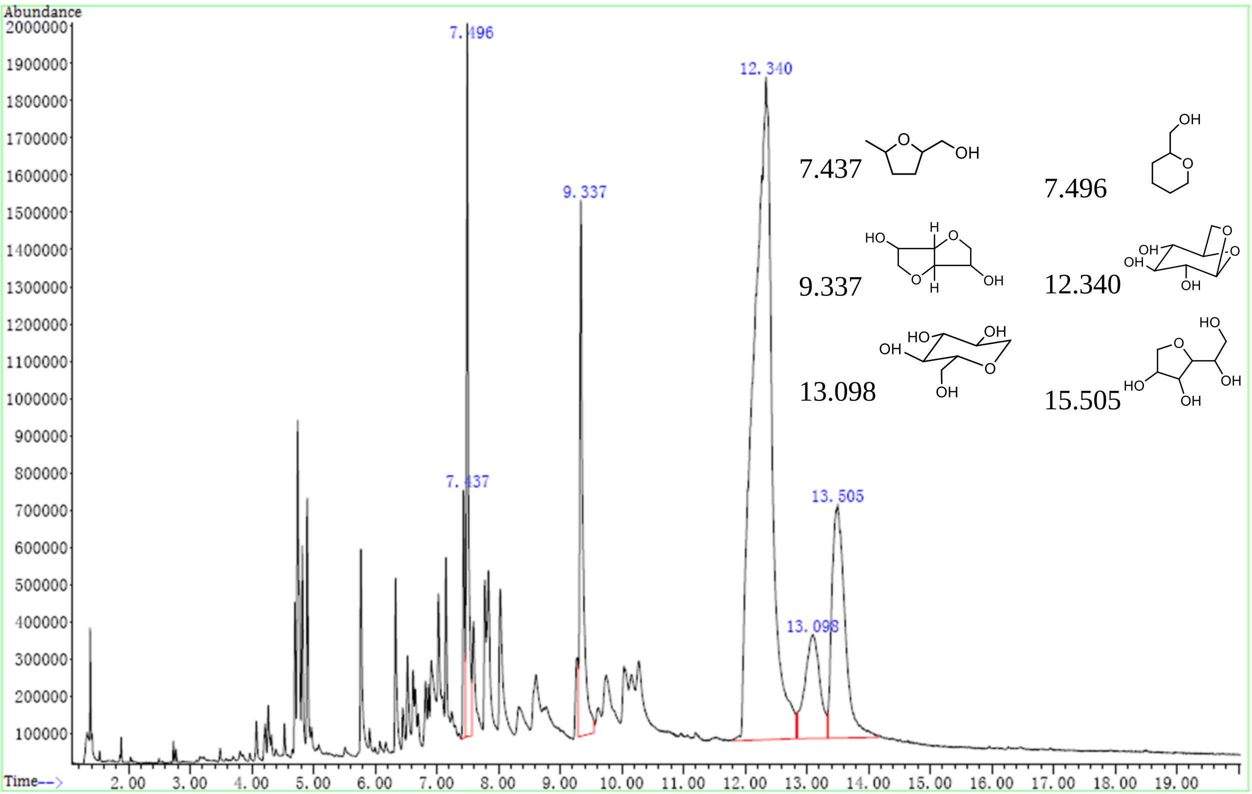

Supplementary Figure 8: GC-MS spectra of reaction effluent obtained after cellobiose reaction at 170 °C for 1 h.

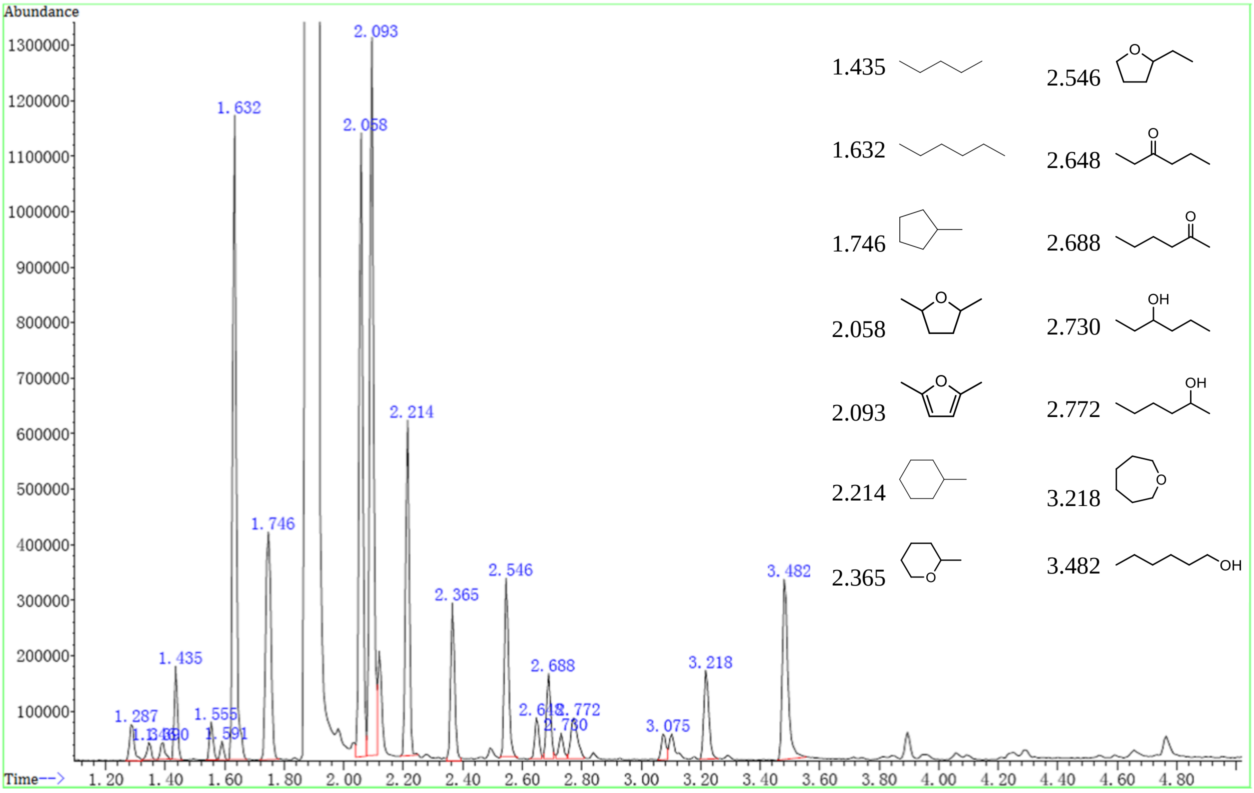

Supplementary Figure 9: GC-MS spectra of organic phase obtained after cellobiose reacting at 170 °C for 6 h.

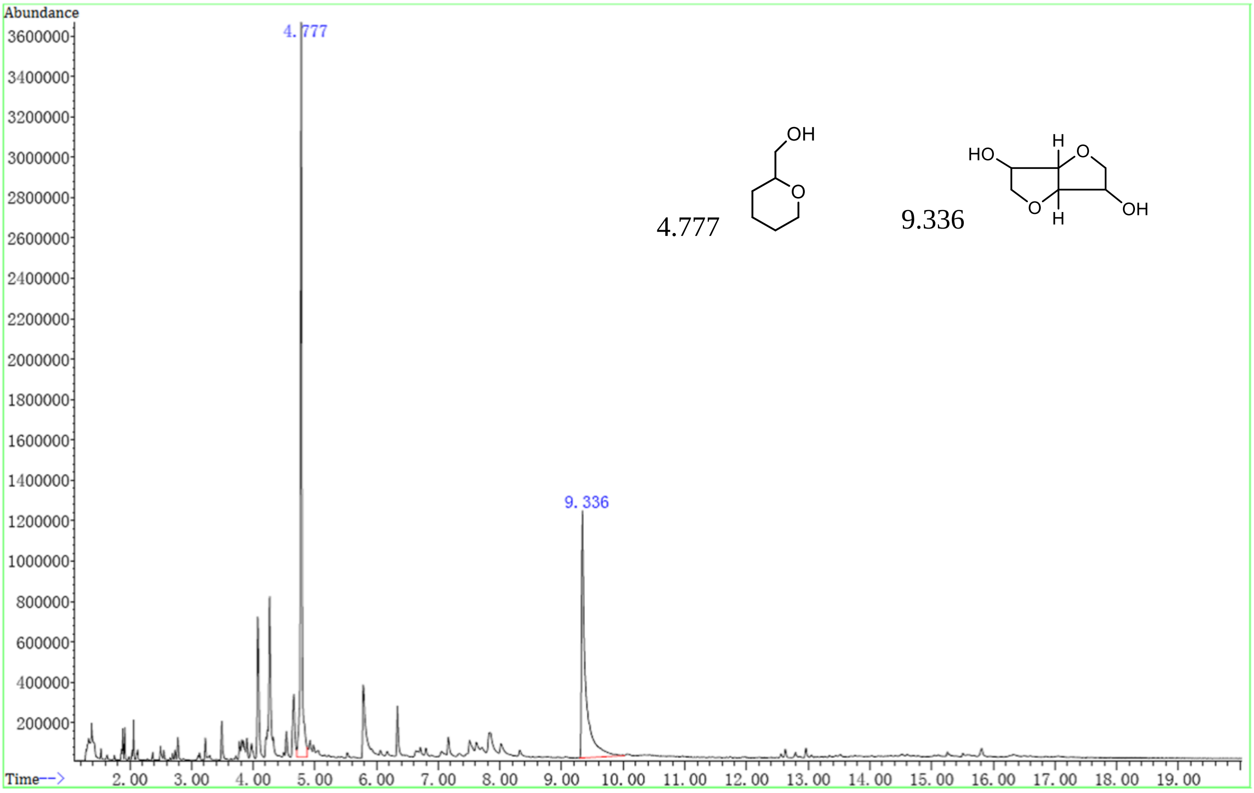

Supplementary Figure 10: GC-MS spectra of aqueous phase obtained after cellobiose reacting at 170 °C for 6h.

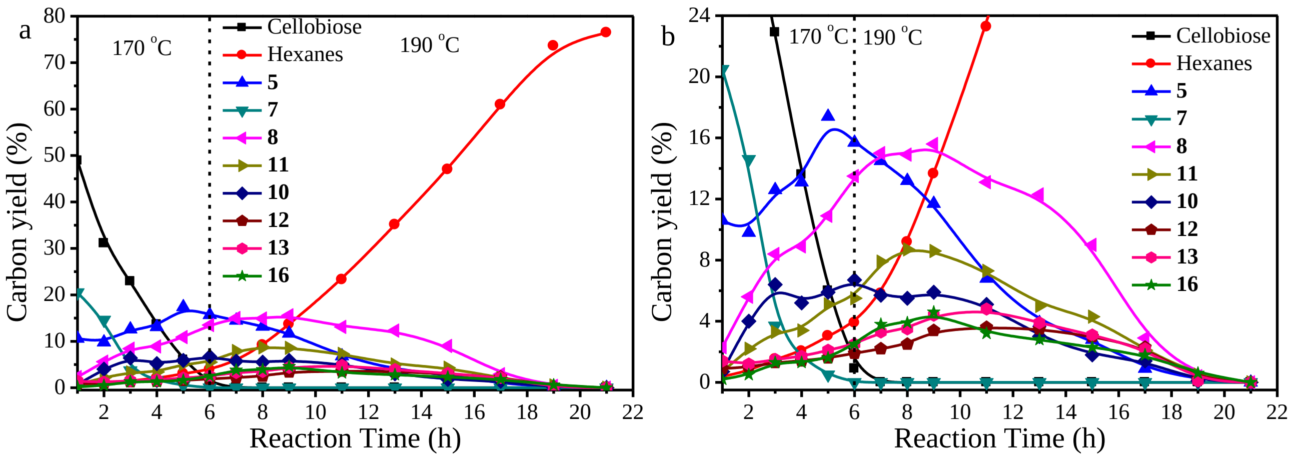

a b

Supplementary Figure 11: The product distribution versus reaction time in the one-pot hydrodeoxygenation of cellobiose in cyclohexane over Pt/NbOPO$_4$ (a) and its enlarged view (b). The reactions were conducted at 170 ^{o}C for 6 h first to better detect the possible intermediates and the reaction temperature was then raised to 190 ^{o}C for another 15 h. The product distribution is very complicated and more than 30 intermediates are observed during the reaction. A number of components show very low concentration that can be hardly determined. Here three major intermediates in aqueous [isosorbide (**5**), 1,6-anhydro-glucose (**7**), 2-hydroxymethyl-tetrahydropyran (**8**)] and five major intermediates in organic phase [2,5-dimethylfuran (**10**), 2,5-dimethyltetrahydrofuran (**11**), 2-methyltetrahydropyran (**12**), 2-ethyltetrahydrofuran (**13**), and n-hexanol (**16**)] are fully quantified. It suggests that the conversion of tetrahydropyran and tetrahydrofuran (THF) derivatives are very slow even at a higher reaction temperature of 190 ^{o}C, confirming that the ring-opening of tetrahydropyran and tetrahydrofuran (THF) derivatives and the subsequent hydrodeoxygenation are the rate-determining steps.

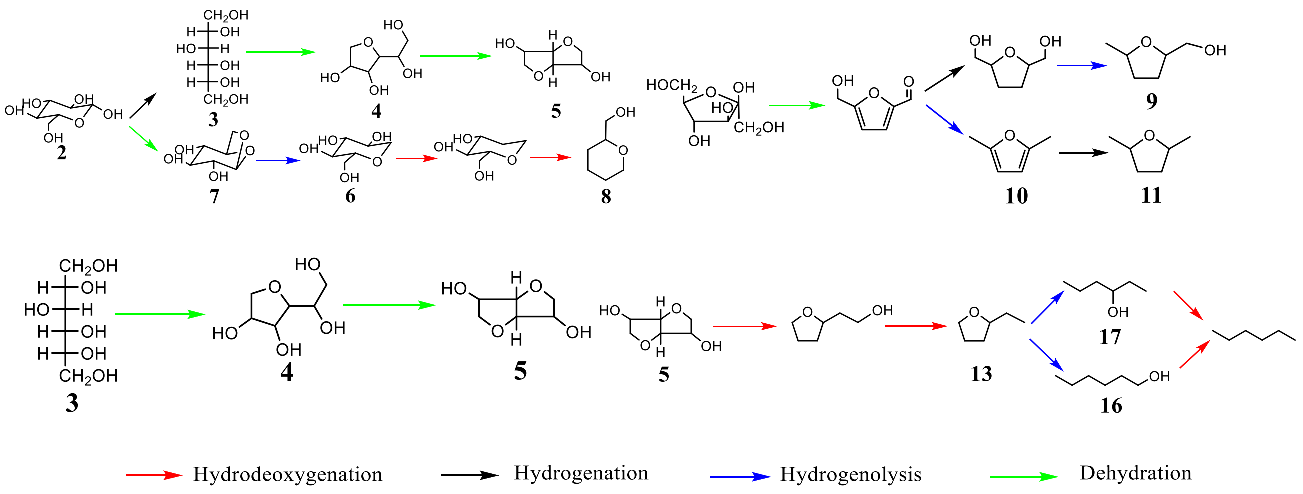

Supplementary Figure 12: The reaction pathways of the isolated reactions using intermediates in cellobiose conversion as feedstock. To further confirm the reaction pathways of cellobiose conversion, isolated reactions were carried out using D-glucose (**2**), fructose, sorbitol (**3**) and isosorbide (**5**) as feedstock, respectively. When reacted for 1 h at 170 ^{o}C with glucose as feedstock, a mixture of **3**, sorbitan (**4**), **5**, 1-dehydroxyl-glucose (**6**), 1,6-anhydro-glucose (**7**) and 2-hydroxymethyl-tetrahydropyran (**8**) were detected in the aqueous phase. The product distribution was very similar to that of cellobiose conversion, suggesting glucose was an early intermediate in cellobiose conversion. With fructose as starting material and reaction for 1 h at 170 ^{o}C, the amount of 2,5-dimethylfuran (**10**), 2,5-dimethyltetrahydrofuran (**11**) and 5-methyl-tetrahydrofuran-2-methanol (**9**) were much higher than that from glucose, indicating that **9**, **10** and **11** were generated from fructose, which can be isomerised from glucose catalysed by Lewis acid sites. With **3** as feedstock under the same reaction condition, the amount of **4** and **5** were much higher than that with glucose as feedstock, suggesting that **4** and **5** were generated from **3**. Then, **5** was employed as feedstock and reaction at 190 ^{o}C for 1 h, a large amount of 2-hydroxyethyl-tetrahydrofuran, 2-ethyltetrahydrofuran (**13**), 3-hexanol (**17**) and 1-hexanol (**16**) as well as n-hexane were detected in liquid phase, confirming the reaction pathway of isosorbide conversion depicted in Figure 2.

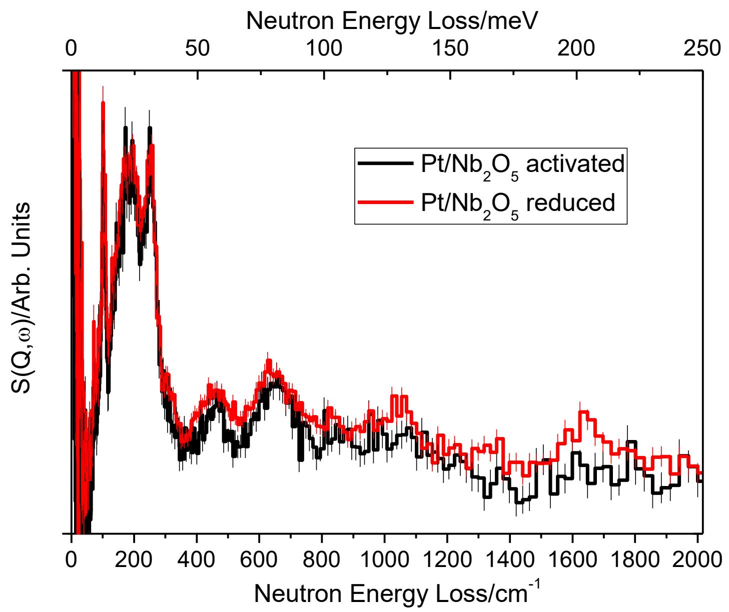

Supplementary Figure 13: Comparison of the INS spectra for activated and reduced catalyst Pt/Nb_2O_5.

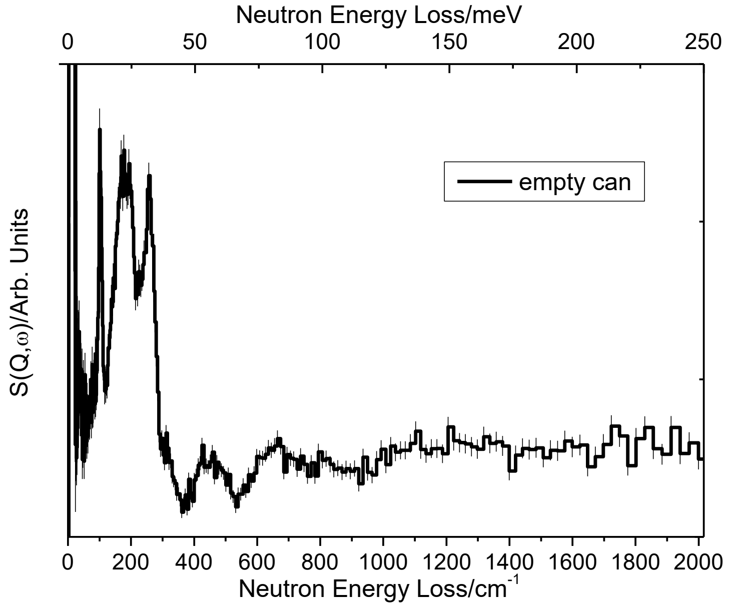

Supplementary Figure 14: View of the INS spectra for the empty catalysis cell used for this experiment.

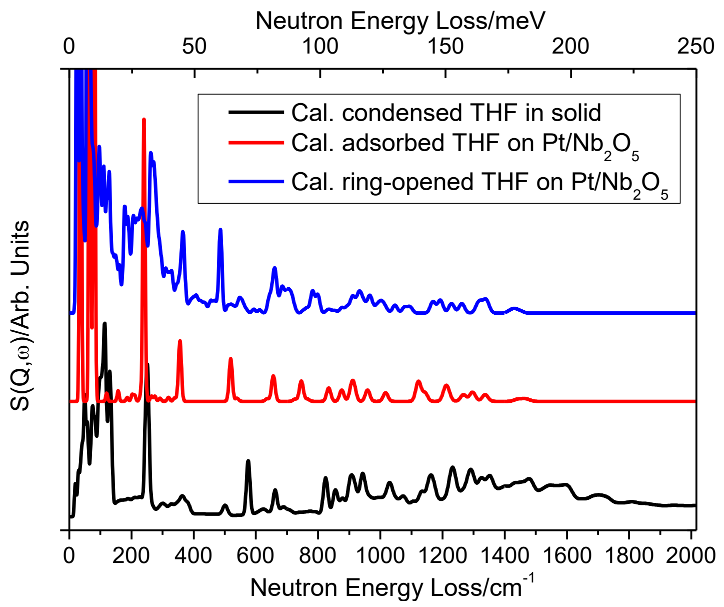

Supplementary Figure 15: Comparison of the DFT calculated INS spectra. Calculated INS spectra for solid THF (bulk phase, black), adsorbed THF (intact molecule, red) on the catalyst surface, and ring-opened THF bound to the surface (blue).

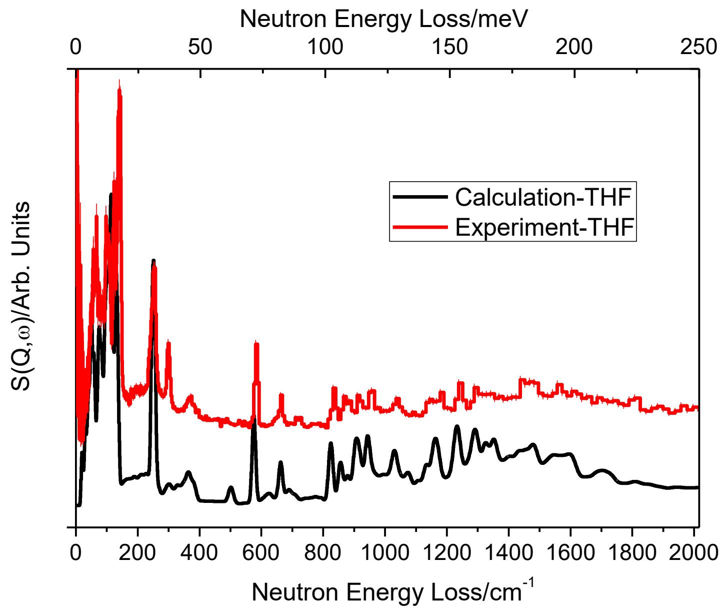

Supplementary Figure 16: **Comparison of the experimental and calculated INS spectra of condensed THF in the solid state.** The calculated INS spectrum shows the total transitions (up to 10 orders).

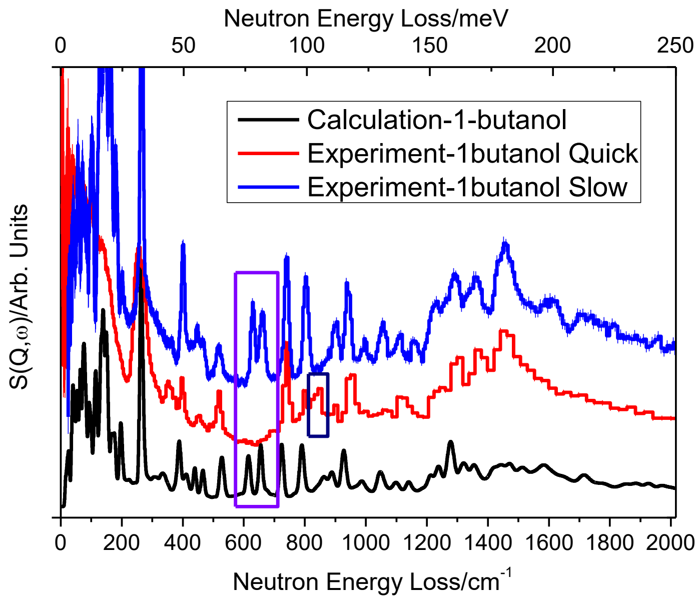

Supplementary Figure 17: Comparison of the experimental and calculated INS spectra of condensed 1-butanol in the solid state. INS spectra for quickly and slowly condensed (liquid to solid) 1-butanol sample are shown in red and blue, respectively. The major differences related to the formation of intermolecular hydrogen bonds are highlighted in boxes.

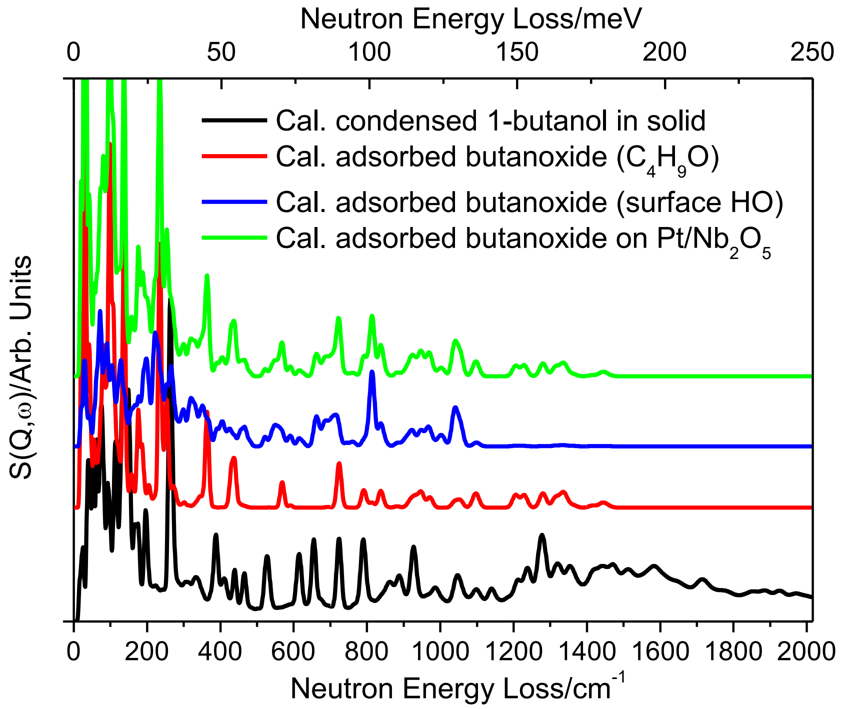

Supplementary Figure 18. Comparison of the DFT calculated INS spectra for solid 1-butanol (bulk phase) and chem-adsorbed 1-butanoxide bound to the surface of the catalyst. The INS spectrum for the latter has contribution from two components: adsorbed C_4H_9O moiety and surface –OH groups, and the vibrational state for each component was also calculated separately and the corresponding INS spectra for adsorbed C_4H_9O moiety and surface –OH groups were generated individually.

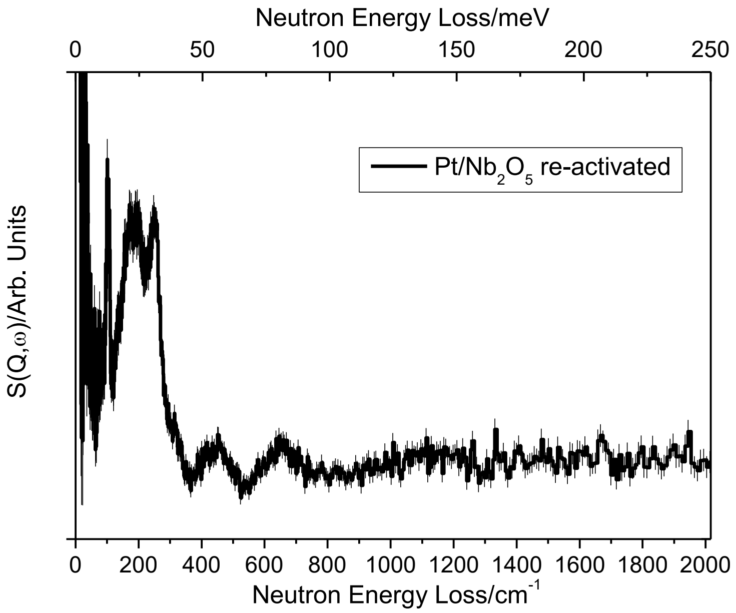

Supplementary Figure 19: View of the INS spectra for the re-activated catalyst Pt/Nb_2O_5 after this experiment.

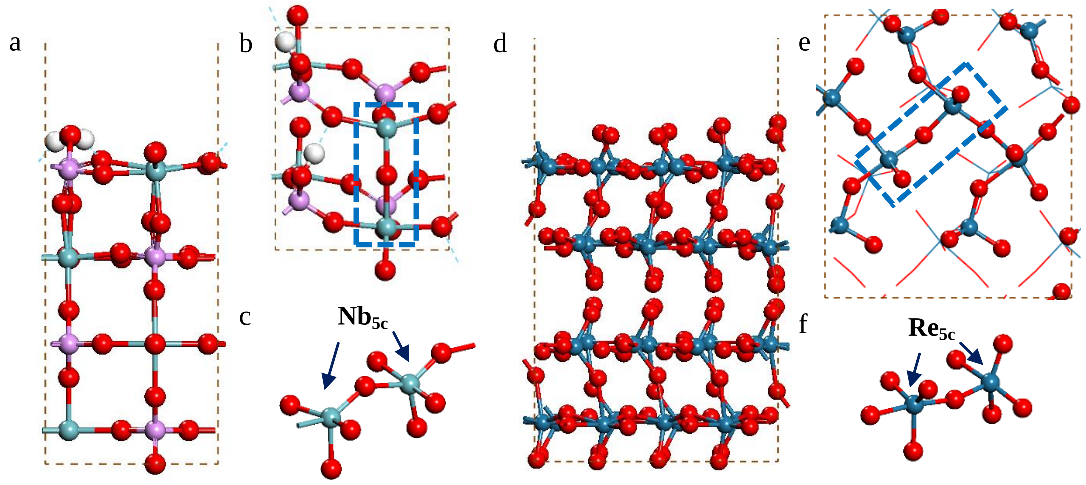

Supplementary Figure 20: Optimised structures of $NbOPO_4$(100) (a, side view; b, top view) and Re_2O_7(010) surface (d, side view; e, top view). On $NbOPO_4$(100), two dangling bond oxygen is saturated with hydrogen. For Re_2O_7(010), two oxygen vacancies were introduced to create two five-coordinated Re (Re_{5c}, in blue rectangular) as active sites. For clarity, the structures of active sites in blue rectangular regions are shown in c and f, respectively. Light blue balls represent Nb atoms, blue for Re, white for H, red for O and pink for P.

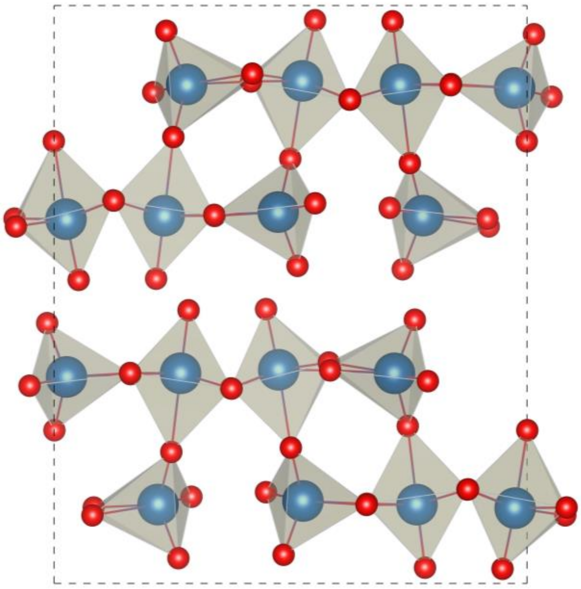

Supplementary Figure 21: The unit cell structure of Re_2O_7. The blue and red spheres represent Re and oxygen atoms, respectively.

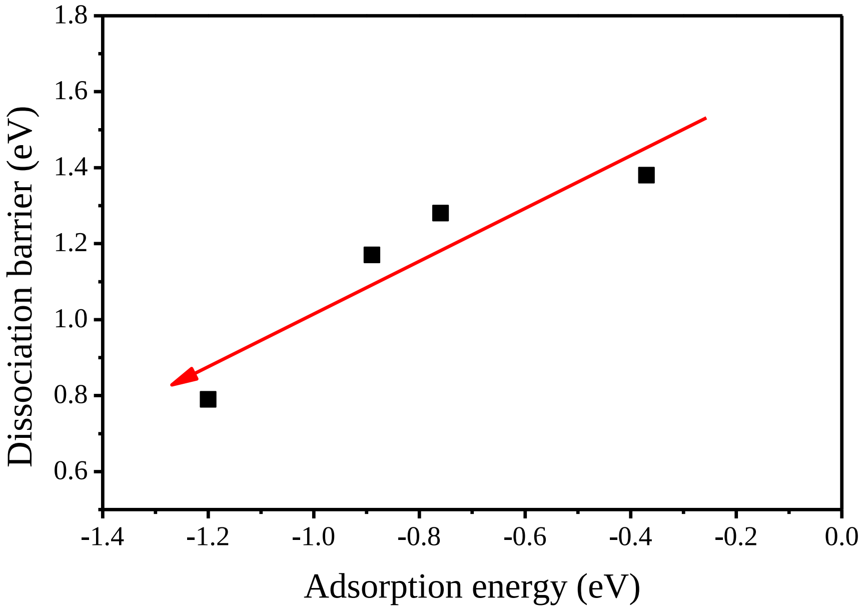

Supplementary Figure 22: Correlation between C-O bond dissociation barriers of butanol/phenol and their corresponding adsorption energies on $NbOPO_4$(100) or Re_2O_7(010) surfaces.

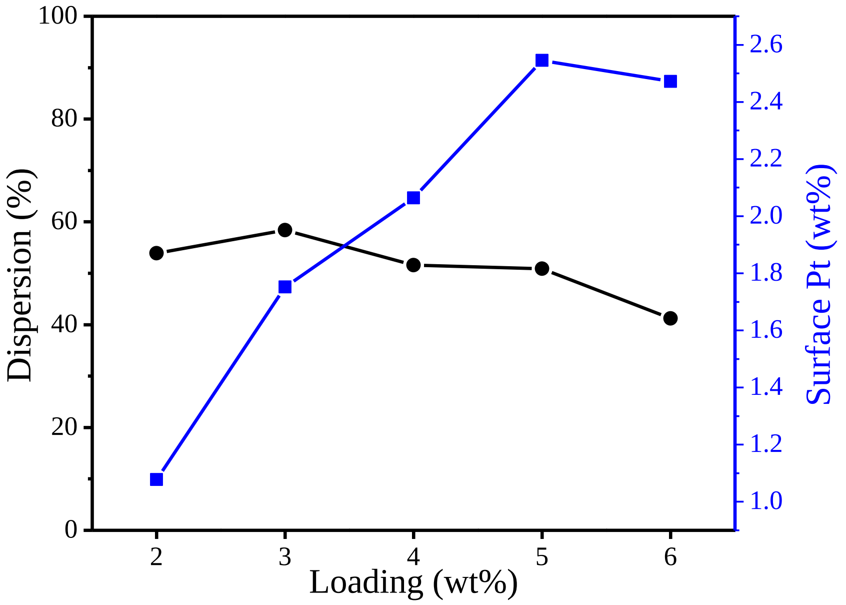

Supplementary Figure 23: The dispersion measurement result of Pt/NbOPO$_4$ catalysts with different Pt loading amount. CO chemisorption for measurement of the dispersion of Pt/NbOPO$_4$ was measured in a chemisorption analyzer (Micromeritics Autochem II 2920) equipped with a thermal conductivity detector (TCD). The samples (100 mg) were loaded into a U-shaped quartz tube. Before CO chemisorption measurements, the catalysts were treated in flowing 10% H_2/Ar mixture at 100 °C for 1 h. Subsequently, the adsorption was performed at 30 °C with pulses of 10.22% CO/He mixture. The volume of the quantitative tube was 0.5173 ml. The number of surface Pt atoms is represented by the adsorption amount of CO assuming that the stoichiometry of adsorbed CO to surface Pt atom is 1.

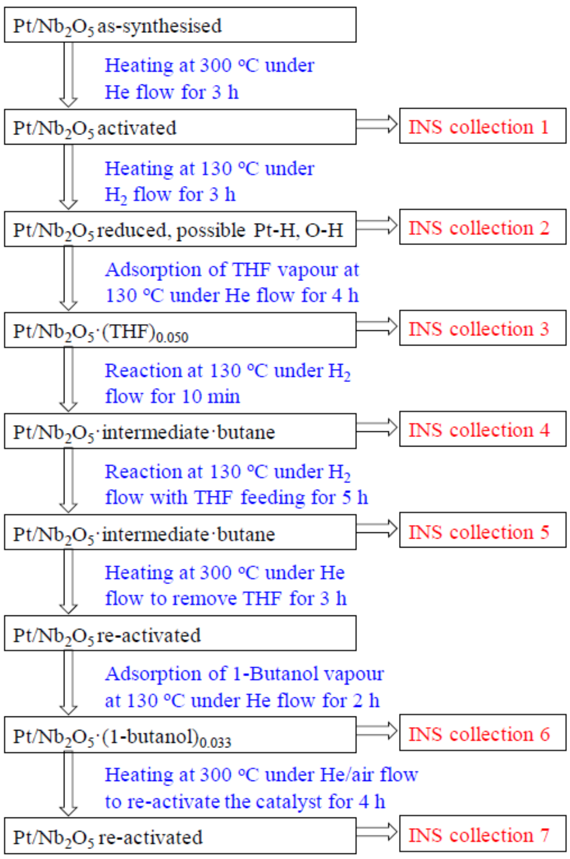

Supplementary Figure 24: Schematic view of the procedure of *in situ* INS experiment and data collection. Seven INS spectra were collected for the catalyst in total.

Supplementary Table 1: Summary of direct hydrodeoxygenation of various feedstocks over Pt/$NbOPO_4$.[a]

Entry	Feedstock	Carbon yield of liquid alkanes[%]		
		Hexanes [b]	Pentanes [c]	alkylcyclohexanes
1	Pine lignin [d]	ND [e]	ND	5.4
2	Birch lignin [d]	ND	ND	12.1
3 [f]	Diphenyl ether	ND	ND	99.9
4 [f]	Phenol	ND	ND	99.9
5	Cellulose	71.5	8.7	ND
6	Cellobiose	76.4	8.9	ND

[a] The reactions were conducted at 190 °C and 5 MPa H_2 for 20 h. Feedstock (0.2 g), catalyst (0.2 g), and cyclohexane (6.46 g) were put into a 50 mL stainless-steel autoclave. [b] Hexanes include n-hexane and iso-hexane. [c] Pentanes include n-pentane and iso-pentane. [d] The lignin was prepared by the following method: Pine wood or birch wood (50g, 40~60 mesh) was suspended in a 300 mL of ethanol-water solution (v/v = 1:1) in a 500 mL autoclave equipped with a mechanical stirrer. The suspension was heated from 25 to 178 °C at 1 °C min^{-1} and processed at 178 °C for 3.3 h under mechanical stirring. Then the mixture was left to cool to room temperature. A reddish-brown solution was obtained after filtering off the wood fibers. The solvent was removed at 40 °C using a rotary evaporator. In sequence, the reddish-brown solid residue was collected and dried under vacuum at 25 °C for 2 days. [e] "ND" is the abbreviation of "not determined". [f] n-Octane was employed as the reaction solvent.

Supplementary Table 2: Activation energies (E_a) and reaction energies (ΔE) of hydrogen dissociation, butane formation and methyl phenol hydrogenation on Pt(111) (0 K).

Adsorption structure	Reaction/hydrogenation	E_a / eV	ΔE / eV
/	$H_2 + 2^* \rightarrow 2H^*$	N/A	-1.02
/	$CH_3CH_2CH_2CH_2^* + H^* \rightarrow$ $CH_3CH_2CH_2CH_3 + 2^*$	0.70	0.05
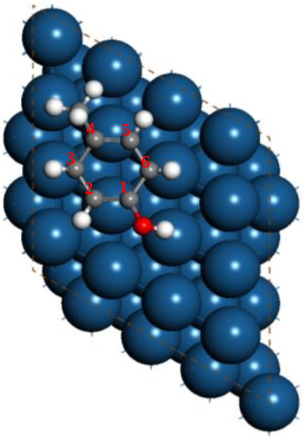	C2	1.14	0.29
	C3	1.06	0.36
	C4	1.04	0.26
	C5	1.08	0.38
	C6	1.17	0.18
	C1	0.86	0.30

Supplementary Table 3: Comparison of catalytic performances of $Pt/NbOPO_4$ with Pt/Nb_2O_5 and $Pd/NbOPO_4$ for hydrodeoxygenation of THF or cellulose.[a]

Entry	Catalyst	Feedstock	Conversion (%)	Carbon yield of alkanes (%)	
				Butane	C_1-C_3 alkanes
1	$Pt/NbOPO_4$	THF	>99.9	94.2	5.8
2	Pt/Nb_2O_5	THF	>99.9	95.8	4.2
3[b]	$Pt/NbOPO_4$	Cellulose	98	71.5	8.7
4[b]	$Pd/NbOPO_4$	Cellulose	98	63.7	7.3

[a] The reactions were conducted at 190 °C and 5 MPa H_2 for 20 h. THF (0.2 g), catalyst (0.1 g), and cyclohexane (6.46 g) were put into a 50 mL stainless-steel autoclave. [b] 0.2 g of catalyst was used.

Supplementary Table 4: Effect of the surface (top layer) H coverage on O adsorption energy (E_{ad}(O))

Coverage	E_{ad}(O) / eV	
	$NbOPO_4$(100)	Re_2O_7(010)
0	0.96	-1.99
1/3	/	-2.70
1/2	-1.60	/
2/3	/	-2.34
1	-4.44	/

Supplementary Table 5: Activation energies (E_a) and reaction energies (ΔE) of C_4H_9OH conversion on $NbOPO_4$(100).

	Reaction	E_a /eV	ΔE /eV
	C_4H_9OH + *→ C_4H_9OH*	N/A	-1.20
Path I	C_4H_9OH* +* → C_4H_9* + OH*	0.79	-1.72
Path II	C_4H_9OH* + O_{latt} → C_4H_9O* + $O_{latt}H$	0.28	-0.18
	C_4H_9O* + * →C_4H_9* + O*	1.48	0.81

Note: In C_4H_9OH* + O_{latt} → C_4H_9O* + $O_{latt}H$ and C_4H_9O* + *→ C_4H_9* + O*, co-adsorption structures for the corresponding product is used to calculate ΔE.

Supplementary Table 6: Calculated binding energies (eV) of some important intermediate species on $NbOPO_4(100)$, $Re_2O_7(010)$ and $ZrO_2(010)$

species	Reaction	$NbOPO_4(100)$	$Re_2O_7(010)$	$ZrO_2(010)$
O	$1/2O_2 + * \rightarrow O*$	-4.44	-1.99	1.43
OH	$H_2O + * \rightarrow OH* + 1/2H_2$	-1.95	-0.13	1.06
H	$1/2H_2 + * \rightarrow H*$	-0.59	0.43	2.17
C_4H_8O	$C_4H_8O + * \rightarrow C_4H_8O*$	-1.15	-0.83	-0.80
C_4H_9OH	$C_4H_9OH + * \rightarrow C_4H_9OH*$	-1.20	-0.76	-0.69
OC_4H_9	$C_4H_9OH + * \rightarrow C_4H_9O* + 1/2H_2$	-2.12	-0.32	1.14
C_4H_9	$C_4H_{10} + * \rightarrow C_4H_9* + 1/2H_2$	-0.09	0.94	1.94

Supplementary Note 1: Calculation of maximum theoretical carbon yield of monomer alkylcyclohexanes

The catalytic hydrogenolysis of C_x-O-C_y linkages results in the formation of a certain amount of monomers, and total hydrodeoxygenation of these resultant monomers produces the monomer hydrocarbons (alkylcyclohexanes). Given the ratio between C_x-O-C_y and C_x-C_y linkages, it is possible to calculate the theoretical yields of monomers[1]. For example, in the case of a hardwood with 70% C_x-O-C_y and 30% C_x-C_y linkages, the maximum theoretical yields of monomers upon selective cleavage of C_x-O-C_y are 49% (70%*70%) of the lignin, and a hardwood with 66.5% C_x-O-C_y and 33.5% C_x-C_y linkages, the maximum theoretical yields of monomers upon selective cleavage of C_x-O-C_y are 44% (66.5%*66.5%) of the lignin.

C_x-O-C_y and C_x-C_y linkages of lignin:

C_x-O-C_y Linkages:

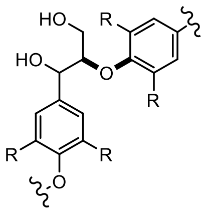

β-O-4

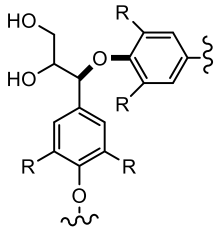

α-O-4

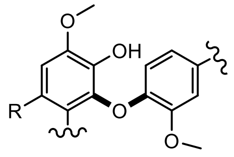

4-O-5

C_x-C_y Linkages:

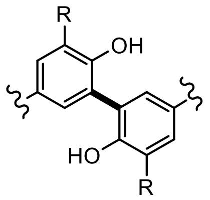

5-5

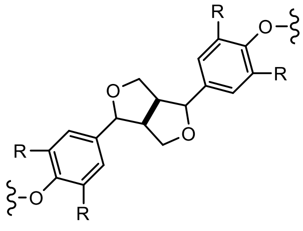

β-β

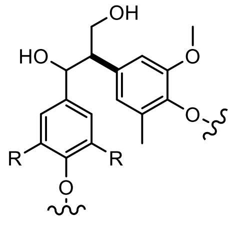

β-1

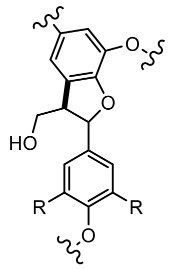

β-5

Three monolignols of lignin:

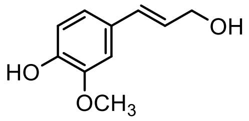

Coniferyl alcohol

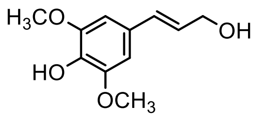

Sinapyl alcohol

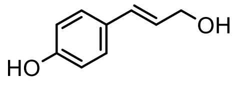

Paracoumaryl alcohol

Supplementary Note 2: Stability tests of the Pt/$NbOPO_4$ catalyst

The recyclability of the Pt/$NbOPO_4$ catalyst was investigated at 190 ^oC and 5 MPa initial H_2 pressure for 8 h using cellulose as feedstock. After each reaction cycle, the catalyst was first separated from the liquid phase by centrifugation, then dried and regenerated for the next run. The XRD patterns of the fresh and spent Pt/$NbOPO_4$ showed that the $NbOPO_4$ support remained amorphous after reaction (Supplementary Fig. 5). The specific BET surface area of Pt/$NbOPO_4$ decreased from 217 m^2 g^{-1} (before reaction) to 156 m^2 g^{-1} after reaction, likely due to the loss of some micropores after the reaction. The CO adsorption measurement showed that the dispersion of Pt on $NbOPO_4$ support decreased from 50.9% to 29.6%, due to the slow growth of the Pt particle and decrease of the specific area of the support. Nevertheless, the decreases of the microporosity and Pt dispersion have little influence on the activity of the catalyst in this reaction within four cycles.

Supplementary Note 3: Comparison of INS and traditional optical spectroscopy

INS was used to study the binding interaction and structure dynamics in this case, because it has several unique advantages:

- INS spectroscopy is sensitive to the vibrations of hydrogen atoms, and hydrogen is ten times more visible than other elements due to its high neutron cross-section.
- The technique is not subject to any optical selection rules. All vibrations are active and, in principle, measurable.
- INS observations are not restricted to the centre of the Brillouin zone (gamma point) as is the case for optical techniques.
- INS spectra can be readily and accurately modelled: the intensities are proportional to the concentration of elements in the sample and their cross-sections, and the measured INS intensities relate straightforwardly to the associated displacements of the scattering atom. Treatment of background correction is also straightforward.
- Neutrons penetrate deeply into materials and pass readily through the walls of metal containers making neutrons ideal to measure bulk properties of this material (in this case for 34.6 g catalyst).
- INS spectrometers cover the whole range of the molecular vibrational spectrum, 0-500 meV (0-4000 cm^{-1})
- INS data can be collected at low temperature (< 15 K in this case), where the thermal motion of the catalyst, the adsorbed THF and the reacted intermediate molecules can be significantly reduced.
- Calculation of the INS spectra from DFT vibrational analysis can be readily achieved, which is directly related to the INS spectra, and in the case of solid state calculations, there are no approximations other than the use of DFT eigenvectors and eigenvalues to determine the spectral intensities.

Supplementary Note 4: Additional discussion of inelastic neutron scattering (INS) studies.

Approximately 35 g of 5% Pt/Nb_2O_5 was loaded into a flow-type stainless steel catalysis cell. Due to the large surface area (100 m^2 g^{-1}) of the catalyst Pt/Nb_2O_5, a small amount of moisture is adsorbed onto the catalyst upon exposure to air at ambient conditions. The catalyst was activated by heating at 300 °C under He flow for 3 hours before the INS experiment to remove the adsorbed water molecules (~0.3 wt%). As expected, the bare catalyst, Pt/Nb_2O_5, gives no prominent features in the measured INS spectra as a result of the low cross-sections of Nb and O for neutron scattering (Supplementary Fig. 12). The features below 300 cm^{-1} and at approximately 457 and 659 cm^{-1} are contributed by the stainless steel catalysis cell as confirmed by comparing to the INS spectra of the empty cell (Supplementary Fig. 13). This result also confirms the complete removal of the adsorbed water molecules from the catalyst.

Upon reduction in H_2 flow at 300 °C for 3 hours, the activated catalyst is partially reduced. Comparison of the INS spectra of activated and reduced catalyst (Supplementary Fig. 12), three small but noticeable peaks (~1045, 1330 and 1638 cm^{-1}) were shown after the reduction in H_2. There are a number of possible explanations for the appearance of hydrogen on the catalyst after reduction: (i) formation of surface Nb-O-H bond; (ii) formation of water molecules from the reduction of the tiny amount of surface PtO_x to Pt(0); (iii) formation of Pt-H bond. The peak at 1045 and 1638 cm^{-1} are consistent with the bending modes of surface Nb-O-H groups and H-O-H in water molecules, respectively, and the quantity of both components are very small as suggested by the low peak intensity. Additionally, the increase in intensity at 600 cm^{-1} is likely due to the librational band of water. The vibrational bands for Pt-H typically occur at 500-800 cm^{-1} and this is not shown clearly in this case, which is mainly due to the low loading of Pt (5 %). These bands are only visible with high Pt loading, typically at ~40%[2]. Owing to the very small increase in intensity of INS spectra, more definitive insight into the structural change of the catalyst after H_2 reduction cannot be obtained.

INS spectra were also collected for condensed solid THF and 1-butanol to assist the identification of their vibrational modes in adsorbed state on the catalyst. The experiment was done by filling liquid THF or 1-butanol into a flat cell (~2.5 ml) which was then sealed and immediately quenched in liquid nitrogen (77 K) and substantially transferred into the CCR cryostat at temperature below 15 K for INS collection. Calculated INS spectra were obtained by DFT based upon their solid state crystal structures as obtained from CCDC database. Supplementary Fig. 15 compares the measured and calculated INS spectra of solid THF, and excellent agreement is obtained. The only differences are (i) the measured peak at 300 cm^{-1} is underestimated in the calculation and (ii) the calculated weak peak at 501 cm^{-1} is not present in the experimental spectrum. The former is likely due to the combination of the lattice mode at ~60 cm^{-1} and the strong mode at 251 cm^{-1}

(assigned to the torsional mode of the C2–C3 bond of the THF ring) in the measured spectrum. The calculated band at 501 cm^{-1} is the first overtone of the 251 cm^{-1} mode. The reason it appears is because the intensity of the fundamental mode at 251 cm^{-1} is over-estimated in the calculation, which is suggested by the relative low intensities of the lattice modes below 150 cm^{-1} and the torsion mode at 251 cm^{-1} in the experimental spectra (Supplementary Fig. 15). It is worth noting that in optical spectroscopy, overtones are formally forbidden and may only appear as weak signals. However, overtones are commonly observed in neutron spectroscopy, and have been accounted for in the calculated spectra[3].

Supplementary Fig. 16 shows the comparison of the measured INS spectrum of 1-butanol with that calculated from its crystal structure. The agreement is, however, poorer than that of THF. In particular, two modes calculated at 614 and 655 cm^{-1} (lower purple box), which are assigned to the out-of-plane C–O–H bending modes, are not seen experimentally and a broad mode observed at 839 cm^{-1} (upper blue box) is not predicted in the calculated INS data. Re-running the DFT calculation with much tighter convergence criteria made no difference to the calculated INS spectra. Inspection of the region below 200 cm^{-1} offers a potential explanation of this discrepancy. The experimental spectrum below 200 cm^{-1} displays a broad continuum with little substructure which is reminiscent of an amorphous solid and 1-butanol is known to undergo a considerable structural reorganisation on crystallisation[4]. In particular, the inter-molecular hydrogen bonds in the solid are much weaker than those in the liquid phase due to the reduction of freedom of external modes (*i.e.*, rotation and translation) on crystallisation. Thus the experimental INS spectrum obtained after rapid cooling has generated a glass that mirrors the strong hydrogen bonding in the liquid, while the calculation is based on crystalline solid with much weaker hydrogen bonding. Increasing hydrogen bonding strength shifts the C–O–H bending modes to higher energy, and thus the calculated bands at 614 and 655 cm^{-1} in the solid shift to higher energy at 839 cm^{-1} (assigned to the C–O–H bending modes in the liquid/glassy state of 1-butanol) with a relatively broad feature. This hypothesis is also evidenced by the relatively good agreement between experimental and calculated INS spectra for the in-phase rocking modes of $-(CH_2)_3-$ chain (737 cm^{-1}), deformation bands (800-1400 cm^{-1}) and the methyl torsion at 258 cm^{-1}, the last of which usually displays considerable INS intensity[3]. To further test this hypothesis, an INS spectrum for 1-butanol that was annealed just below the melting point (183 K) to generate a crystalline solid was measured (Supplementary Fig. 16). Much improved agreement between experiment and calculation is observed, confirming the assignment of the bands participating in the hydrogen bond formation.

The adsorption of 1-butanol on the catalyst was studied because 1-butanoxide bound to Nb^{5+} is predicted to be relatively stable during the hydrodeoxygenation (Figure 3i). The INS spectrum of 1-butanol adsorbed on the

catalyst shows a significant increase in total intensity (Figure 3c). Comparison of the difference spectrum and that of condensed 1-butanol in solid shows a few changes (Figure 3f). The bands between 300 and 600 cm^{-1} in the INS spectrum of solid 1-butanol all disappeared upon adsorption. These bands are conformation dependent and indicate that the alkyl chain of adsorbed 1-butanol is not in the all-*trans* conformation as found in crystalline 1-butanol. There is reasonable agreement with the spectrum generated from the DFT optimised model (Figure 3i), which has a *gauche* conformation for the C4 chain, consistent with the experimental data. Indeed, 1-butanol has several conformers that are close in energy[5] and it is likely that more than one conformer is present on the surface of the catalyst. The DFT-optimised model also has a surface hydroxyl (Figure 3i), and the calculation indicates that this is strongly hydrogen bonded and has out-of-plane O-H modes at 814 and 1042 cm^{-1}. This is consistent with the presence of the peak at 873 cm^{-1} and a slight increase in intensity at 1044 cm^{-1} in the experimental spectrum (Figure 3f), though the calculation slightly underestimates the strength of hydrogen bonding. Under the high temperature and flow conditions used to adsorb 1-butanol to the catalyst, most of the hydroxyls probably underwent further reaction to water, desorbed and were swept out of the cell, the presence of water was confirmed by mass spectrometry.

A final INS spectrum was collected for the re-activated catalyst after the reaction by heating at 300 °C for 4 hours (Supplementary Fig. 17). A clean background with no prominent feature is seen in comparison to the INS spectrum of the empty cell (Supplementary Fig. 13), confirming the complete removal of the organic molecules and reacted species. This result also confirms that no hydrocarbonaceous species was formed on the catalyst and thus demonstrating the high efficiency of the re-generation of the catalyst in cycling experiments.

Supplementary Note 5: Details of C-O bond cleavage of butanol and phenol

On a $NbOPO_4$(100) surface, 1-butanol can efficiently adsorb at Nb_{5c} through its O-end with the Nb-O bond length being at 2.205 Å (Figure 5a). The corresponding adsorption energy was calculated to be as high as -1.20 eV, which is evidently larger than that on Re_2O_7(010) (-0.76 eV), indicating that $NbOPO_4$(100) possesses a better binding ability. With the help of the surface Nb_{5c} site, the C-O bond of the adsorbed butanol can be broken *via* a radical-like transition state structure (TS1 in Figure 5a), leading to a low dissociation barrier of 0.79 eV. In TS1, the C-O bond of 1-butanol is elongated to 2.00 Å from the original 1.46 Å, and the butyl group assumes a radical-like state without evident chemical bonds with the surface Nb_{5c}. More importantly, this whole dissociation process is exothermic by 1.72 eV, with the dissociated hydroxyl and butyl group occupying each Nb_{5c} site as the product. From the energy profile shown in Figure 5a, we can see that the whole deoxygenation process can occur relatively easily on $NbOPO_4$(100).

It is worth mentioning that another possible C-O bond cleavage pattern *via* butoxy (C_4H_9O) dissociation on $NbOPO_4$(100), which is produced from C_4H_9OH dehydrogenation, was also tested here. As for the C_4H_9OH dehydrogenation on clean $NbOPO_4$(100), DFT calculations indicate that it can easily proceed to form C_4H_9O at the initial stage, with its H in hydroxyl strongly bonded to the exposed one-coordinated O in phosphate ion (PO_4^{3-}); however, it will accordingly result in the fast saturation of PO_4^{3-} by H and limit the further dehydrogenation. Alternatively, we considered C_4H_9OH dehydrogenation through the two-coordinated bridge lattice O (in –Nb-O-Nb-) on the more realistic H-saturated $NbOPO_4$(100). As shown in Supplementary Table 5, it corresponds to a low barrier (0.28 eV) but very weak energy release (-0.12 eV), implying the approximate conversion equilibrium between C_4H_9OH and C_4H_9O; in other words, C_4H_9O and C_4H_9OH can coexist, and C_4H_9O has a relatively larger coverage. Nevertheless, the dissociation barrier of C-O bond in C_4H_9O is much increased to 1.48 eV, nearly twice as high as that in the direct dissociation of 1-butanol (0.79 eV). Comparing these two C-O cleavage pathways (Path I and Path II in Supplementary Table 5) starting from the adsorbed C_4H_9OH, the effective barriers are 0.79 eV and 1.30 eV, respectively, implying that C-O bond cleavage of 1-butanol should occur more readily through the direct molecular dissociation (Path I). The inherent origin of such an activity difference (Path I versus Path II) could be largely ascribed to the five-coordinated configuration of the surface Nb_{5c} (the active site), which can form only one single bond in principle, resulting in easier accommodation of OH species rather than the highly unsaturated O atom.

Similar examination of the Re_2O_7(010) surface indicated that the C-O activation barrier of butanol is 1.28 eV, much higher than on $NbOPO_4$(100), and the corresponding transition state (TS1') structure (Figure 5a) was shown to exhibit a longer C-O bond length (2.51 Å). Much worse, this dissociation process is endothermic by

0.12 eV on Re_2O_7(010). By comparing the energy profiles, one can see that $NbOPO_4$ catalysts present better performance for 1-butanol deoxygenation than Re_2O_7 catalysts both thermodynamically and kinetically.

To examine the catalytic activity of $NbOPO_4$ in promoting C-O bond cleavage of lignin, here the deoxygenation process of phenol serving as the model compound is also briefly investigated. Similar to 1-butanol, phenol prefers to adsorb at the five-coordinated Nb_{5c} site on $NbOPO_4$(100) with the O-end, giving an adsorption energy of -0.89 eV, while it is only -0.37eV for Re_2O_7(010) (Figure 5b). The adsorbed phenol can open its C-O bond through a radical like mechanism with a C-O bond distance of 1.98 Å in the transition state structure (TS2 in Figure 5b), corresponding to a moderate barrier of 1.17 eV. In the dissociation product, the hydroxyl and phenyl adsorb at one Nb_{5c} site, respectively (insert in Figure 5b), corresponding to a reaction enthalpy of -1.66 eV. Thus, it confirmed that $NbOPO_4$ should also be able to convert phenol into alkane. On the contrary, the adsorption energy of phenol on Re_2O_7(010) was much weaker at -0.37 eV, while the subsequent dissociation is endothermic by 0.05 eV and the reaction barrier is also higher (1.38 eV, TS2' in Figure 5b). Therefore, similar to the situation of 1-butanol, $NbOPO_4$(001) shows evident advantages in dissociating phenol when compared to the Re_2O_7(010) surface.

Supplementary Methods

Materials. Pine, white pine, larch, fir, camphor, birch and poplar wood sawdusts were obtained locally. They were dried at 120 °C for 2 days, ball-milled for 4 h and sieved by 200-mesh sieves before use. The components (extracts, cellulose, hemicellulose, lignin and ash) of wood sawdusts were analysed according to the procedures of the Van Soest method[6]. H-ZSM-5 (Si/Al=50), SiO_2 and activated carbon were purchased commercially. All the other chemicals were of analytic grade and used directly without further purification.

DFT Calculations and modelling of the INS spectra. Periodic density functional theory (periodic-DFT) calculations were carried out using the plane wave pseudopotential method as implemented in the CASTEP code[7,8]. Exchange and correlation were approximated using the PBE functional[9]. The plane-wave cut-off energy was 830 eV. Brillouin zone sampling of electronic states was performed on 4×4×1 Monkhorst-Pack grid. The equilibrium structure, an essential prerequisite for lattice dynamics calculations was obtained by BFGS geometry optimization after which the residual forces were converged to zero within 0.012 eV $\cdot$A^{-1}. Phonon frequencies were obtained by diagonalisation of dynamical matrices computed using density-functional perturbation theory[10]. The atomic displacements in each mode that are part of the CASTEP output, enable visualization of the modes to aid assignments and are also all that is required to generate the INS spectrum using the program ACLIMAX[11]. It is emphasised that for all the calculated spectra shown the transition energies have not been scaled.

Density functional theory calculation. All the spin-polarised calculations were performed with the Perdew-Burke-Ernzerhof (PBE) functional within the generalised gradient approximation as implemented in the VASP package[12,13]. The project-augmented wave (PAW) method was used to represent the core-valence electron interaction[14]. To model the $NbOPO_4$(100) surface, a four-layer $p(1\times2)$ slab (6.463×8.286 Å^2, 56 atoms, Supplementary Fig. 20a, b) was used, in which 3×3×1 k-point sampling was applied. The bulk structure of Re_2O_7 consists of alternating octahedral and tetrahedral Re centres (Supplementary Fig. 21), and thus a four-layer $p(2\times1)$ slab (10.896×12.508 Å^2, 142 atoms, Supplementary Fig. 20d, e) was used to model the Re_2O_7(010) surface, in which two oxygen vacancies were introduced to create two five-coordinate Re (Re_{5c}, in blue rectangular, Supplementary Fig. 20e, f) as active sites. Because of the large size of the Re_2O_7(010) slab, a Monkhorst Pack mesh with Γ point k-point sampling in the surface Brillouin zone was used, which has been tested to meet the accuracy requirement. The singly coordinated O atoms on bulk-truncated $NbOPO_4$(100) surface were saturated with H atoms to simulate the hydrogen-rich atmosphere; while for Re_2O_7(010), the effect of hydrogen was also tested, which shows relatively small effects on the adsorption energy of O and OH (Supplementary Table 4). Thus, singly coordinated O atoms (6 O atoms) at Re_2O_7(010) surface were not

saturated with hydrogen for saving computing time. A vacuum layer of 15 Å was applied and the top layer was allowed to fully relax for both surfaces. For total energy calculations, the valence electronic states were expanded in plane wave basis sets with a cutoff energy of 450 eV. Atomic positions were relaxed until the maximum force of each atom was less than 0.05 eV/Å. The transition states were searched for using a constrained optimisation scheme[15] and were verified when (i) all forces on the atoms vanish and (ii) the total energy is a maximum along the reaction coordinate but a minimum with respect to the rest of the degrees of freedom.

Supplementary References

1 Azadi, P. *et al.* Catalytic conversion of biomass using solvents derived from lignin. *Green Chemistry* **14**, 1573, (2012).

2 Parker, S. F. *et al.* Characterisation of the adsorption sites of hydrogen on Pt/C fuel cell catalysts. *Catalysis Today* **114**, 418-421, (2006).

3 Mitchell, P. C. H., Parker, S. F., Ramirez-Cuesta, A. J. & Tomkinson, J. *Vibrational Spectroscopy with Neutrons, with Applications in Chemistry, Biology, Materials Science, and Catalysis*. (World Scientific, Singapore, 2005).

4 Derollez, P., Hedoux, A., Guinet, Y., Danede, F. & Paccou, L. Structure determination of the crystalline phase of n-butanol by powder X-ray diffraction and study of intermolecular associations by Raman spectroscopy. *Acta Crystallographica Section B-Structural Science* **69**, 195-202, (2013).

5 Ohno, K., Yoshida, H., Watanabe, H., Fujita, T. & Matsuura, H. Conformational Study of 1-Butanol by the Combined Use of Vibrational Spectroscopy and ab Initio Molecular Orbitol Calculations. *Journal of Physical Chemistry* **98**, 6924-6930, (1994).

6 Goering, H. K. & Van Soest, P. J. *Forage fiber analyses (apparatus, reagents, procedures, and some applications)*. 1-20 (Agriculture Research Service, USDA, 1970).

7 Clark, S. J. *et al.* First principles methods using CASTEP. *Zeitschrift Fur Kristallographie* **220**, 567-570, (2005).

8 Refson, K., Tulip, P. R. & Clark, S. J. Variational density-functional perturbation theory for dielectrics and lattice dynamics. *Physical Review B* **73**, (2006).

9 Perdew, J. P., Burke, K. & Ernzerhof, M. Generalized gradient approximation made simple. *Physical Review Letters* **77**, 3865-3868, (1996).

10 Milman, V. *et al.* Structural, electronic and vibrational properties of tetragonal zirconia under pressure: a density functional theory study. *Journal of Physics-Condensed Matter* **21**, (2009).

11 Ramirez-Cuesta, A. J. aCLIMAX 4.0.1, The new version of the software for analyzing and interpreting INS spectra. *Computer Physics Communications* **157**, 226-238, (2004).

12 Kresse, G. & Furthmuller, J. Efficiency of ab-initio total energy calculations for metals and semiconductors using a plane-wave basis set. *Computational Materials Science* **6**, 15-50, (1996).

13 Kresse, G. & Furthmuller, J. Efficient iterative schemes for *ab initio* total-energy calculations using a plane-wave basis set. *Physical Review B* **54**, 11169-11186, (1996).

14 Kresse, G. & Joubert, D. From ultrasoft pseudopotentials to the projector augmented-wave method. *Physical Review B* **59**, 1758-1775, (1999).

15 Alavi, A., Hu, P. J., Deutsch, T., Silvestrelli, P. L. & Hutter, J. CO oxidation on Pt(111): An ab initio density functional theory study. *Physical Review Letters* **80**, 3650-3653, (1998).
